# Quantitative and Qualitative Stem Rust Resistance Factors in Barley Are Associated with Transcriptional Suppression of Defense Regulons

**DOI:** 10.1371/journal.pgen.1002208

**Published:** 2011-07-28

**Authors:** Matthew J. Moscou, Nick Lauter, Brian Steffenson, Roger P. Wise

**Affiliations:** 1 Bioinformatics and Computational Biology Graduate Program, Iowa State University, Ames, Iowa, United States of America; 2 Department of Plant Pathology and Microbiology, Iowa State University, Ames, Iowa, United States of America; 3 Center for Responses to Environmental Stresses, Iowa State University, Ames, Iowa, United States of America; 4 Corn Insects and Crop Genetics Research, Agricultural Research Service, United States Department of Agriculture, Iowa State University, Ames, Iowa, United States of America; 5 Department of Plant Pathology, University of Minnesota, St. Paul, Minnesota, United States of America; University of California Davis, United States of America

## Abstract

Stem rust (*Puccinia graminis* f. sp. *tritici*; *Pgt*) is a devastating fungal disease of wheat and barley. *Pgt* race TTKSK (isolate Ug99) is a serious threat to these Triticeae grain crops because resistance is rare. In barley, the complex *Rpg-TTKSK* locus on chromosome 5H is presently the only known source of qualitative resistance to this aggressive *Pgt* race. Segregation for resistance observed on seedlings of the Q21861 × SM89010 (QSM) doubled-haploid (DH) population was found to be predominantly qualitative, with little of the remaining variance explained by loci other than *Rpg-TTKSK*. In contrast, analysis of adult QSM DH plants infected by field inoculum of *Pgt* race TTKSK in Njoro, Kenya, revealed several additional quantitative trait loci that contribute to resistance. To molecularly characterize these loci, Barley1 GeneChips were used to measure the expression of 22,792 genes in the QSM population after inoculation with *Pgt* race TTKSK or mock-inoculation. Comparison of expression Quantitative Trait Loci (eQTL) between treatments revealed an inoculation-dependent expression polymorphism implicating *Actin depolymerizing factor3* (within the *Rpg-TTKSK* locus) as a candidate susceptibility gene. In parallel, we identified a chromosome 2H *trans*-eQTL hotspot that co-segregates with an enhancer of *Rpg-TTKSK*-mediated, adult plant resistance discovered through the Njoro field trials. Our genome-wide eQTL studies demonstrate that transcript accumulation of 25% of barley genes is altered following challenge by *Pgt* race TTKSK, but that few of these genes are regulated by the qualitative *Rpg-TTKSK* on chromosome 5H. It is instead the chromosome 2H *trans*-eQTL hotspot that orchestrates the largest inoculation-specific responses, where enhanced resistance is associated with transcriptional suppression of hundreds of genes scattered throughout the genome. Hence, the present study associates the early suppression of genes expressed in this host–pathogen interaction with enhancement of *R*-gene mediated resistance.

## Author Summary

An important step in molecular plant pathology is the identification of the biologically relevant events that are directly involved in mediating resistance to pathogens. Historically, it is known that *de novo* and modulated gene expression are important components of the immune response. And yet, how exactly regulatory cascades orchestrate transcriptional responses to influence immunity remains unexplored. Several molecular tools have enabled the dissection of the defense transcriptome. One such technique, expression Quantitative Trait Locus (eQTL) analysis, provides the opportunity to identify genes involved in transcriptional regulation and simultaneously identifying their downstream targets. This paper describes an eQTL analysis of a barley population segregating for qualitative and quantitative immunity to stem rust (*Puccinia graminis* f. sp. *tritici*), a devastating pathogen of Triticeae grain crops. Analysis of treatment-specific effects identified several regulatory loci that alter the expression of many inoculation responsive genes. On chromosome 2H, a *trans*-eQTL hotspot coincides with an enhancer of adult plant resistance. Notably, the resistance allele for this locus is associated with suppressing the transcription for hundreds of genes, with some of these having been previously associated with plant disease defense. In this respect, conventional wisdom is challenged by these findings.

## Introduction

Plants respond to invading pathogens with several forms of defense, ranging from the generation of toxic chemicals to programmed cell death [1]. These defense strategies do not occur coincidentally, but rather by successive rounds of chemical, physical, and enzymatic barriers introduced to impede pathogen progression. Initially, pathogen-associated molecular patterns (PAMPs) are recognized by pattern recognition receptors (PRR), which in turn, trigger non-specific defense cascades, also known as PAMP-triggered immunity (PTI) [1], [2]. Generally, these non-specific defense mechanisms successfully block pathogen entry. When these primary impediments fail, a more extreme form of defense known as gene-for-gene resistance, or effector triggered immunity (ETI), may occur if the plant encodes an appropriate resistance (R) protein that recognizes, either directly or indirectly, its corresponding pathogen effector [1], [3]. Though extreme, ETI-mediated programmed cell death restricts pathogen ingress, essentially destroying the nutrient source required for colonization of biotrophic fungi. The translocation of several host R proteins into the nucleus after recognition of cognate pathogen effectors has implicated the regulation of gene expression in ETI [4].

Stem rust, caused by the obligate fungal biotroph *Puccinia graminis*, has been a serious problem wherever wheat and barley are grown [5]–[8]. Urediniospores of *P. graminis* germinate within 4 to 8 hours after inoculation (HAI) during nights with dew formation or rainfall [9]. After germ tube extension and recognition of stomatal openings, appressoria form around 12 HAI. Growth continues, with the generation of a penetration peg that initiates sub-stomatal invagination of host tissue, development of infection hyphae, and differentiation of haustorial mother cells. In barley, penetration into the sub-stomatal space coincides with activation of the defense response (12–24 HAI) [10], [11]. Recognition of the pathogen will occur in the presence of *Rpg* (*Resistance to P*. *graminis*) genes, which mediate resistance to particular *formae speciales* of *P. graminis* by [12]. To date, eight *Rpg* genes have been identified, with five specifying resistance to races of *P*. *graminis* f. sp. *tritici* (*Pgt*) and three to *P*. *graminis* f. sp. *secalis* (*Pgs*) [12]. The identification of a new highly virulent race of *Pgt* known as TTKSK, (commonly referred to as Ug99), initiated a major collaboration to identify resistance genes in germplasm repositories of wheat and barley (www.globalrust.org) [13]-[15]. In a search for loci that mediate resistance to *Pgt* race TTKSK, Steffenson and colleagues identified the *Rpg*-*TTKSK* locus on the long arm of chromosome 5H, contributed by the barley cv. Q21861 [12]. This locus had previously been implicated in stem rust resistance by the fine mapping and cloning of *rpg4* and *Rpg5,* respectively [16]. The recessive resistance gene *rpg4* confers immunity to *Pgt* race QCCJ, while *Rpg5* provides dominant/semi-dominant resistance to *Pgs* isolate 92-MN-90. Sequencing of the genomic region in cv. Morex (genotype  =  *Rpg4; rpg5*) found five candidate genes encoding two nucleotide-binding site (NBS), leucine-rich repeat (LRR) proteins, two actin depolymerizing factors (ADF2, ADF3), and a protein phosphatase 2C protein (PP2C) [16]. *Rpg5* co-segregated with the two NBS-LRR, ADF3, and PP2C encoding genes in the susceptible cv. Morex [16]. Sequencing of resistant cv. Q21861 identified major structural polymorphisms in one of the NBS-LRRs, such that it encoded a unique combination of NBS and LRR domains coupled to a serine/threonine kinase (S/TPK) domain [16]. Virus-induced gene silencing and allele sequencing implicated this NBS-LRR-S/TPK as *Rpg5*. The recessive resistance gene *rpg4* has been associated with *Adf2* by allele and recombinant sequencing [16]. Interestingly, resistance to *Pgt* race QCCJ in the informative recombinants indicates that both *Rpg5* and *rpg4* may be required to mediate an effective resistance response [17]. It is unknown which gene underlies *Rpg-TTKSK* mediated resistance to *Pgt* race TTKSK, but it is hypothesized that both *Rpg5* and *rpg4* are required [12].

Recently, several studies have exploited natural variation combined with expression profiling to decipher complex regulatory pathways, and in some cases phenotypic consequences [18]. This approach is referred to as genetical genomics or expression quantitative trait locus (eQTL) analysis [19], [20]. Invariant to the organism studied, two types of heritable variation have been identified for gene expression in segregating populations; the most predominant form being linked to local variation near the physical position of genes (*cis*-eQTL) and the weaker distant regulation generated by genes that impact the transcriptional status of other genes (*trans*-eQTL) [21]–[23]. Although *trans*-eQTL tend to have more moderate effects than *cis*-eQTL, the functional polymorphisms that permit their discovery often affect more than one gene. For example, if a polymorphism altered activity of a transcription factor or hormone signaling gene, eQTL analysis may trace the regulation of many to hundreds of genes to the locus harboring this polymorphism, which would then be termed a *trans*-eQTL hotspot [24]. One such *trans*-eQTL hotspot affecting secondary metabolism has been associated with the *AOP* (*ALKENYL HYDROXALKYL PRODUCING*) locus, where *cis*-eQTL in genes involved in glucosinolate biosynthesis lead to the altered transcriptional and metabolic status of Arabidopsis [25].

To gain insight into the regulatory functions of the *Rpg-TTKSK* locus and the polymorphism(s) responsible for its existence, we analyzed the mRNA abundance of 22,792 host genes in each member of the Q21861 × SM89010 (QSM) doubled-haploid mapping population subjected to *Pgt* race TTKSK-inoculation (INOC) and mock-inoculation (MOCK). By integrating the dynamics of eQTL hotspot formation, inoculation-responsive gene expression, and alternative control of eQTL between INOC and MOCK treatments, we describe two forms of transcriptional regulation that are associated with the resistance response. First, we provide evidence for *Adf3* (within the *Rpg*-*TTKSK* locus) as a candidate susceptibility gene based on a strong *cis*-eQTL that has its effect magnified by inoculation with *Pgt* race TTKSK. Second, we report the identification of an inoculation-dependent, *trans*-eQTL hotspot that governs the expression of hundreds of genes, which under normal conditions, are controlled by additional modular regulators. The position of this chromosome 2H *trans-*eQTL hotspot is coincident with a quantitative resistance factor that acts as an enhancer of *Rpg-TTKSK*-mediated resistance in adult plants. Notably, the alleles across this shared genomic position that enhance *Rpg-TTKSK*-mediated resistance, lead to transcriptional suppression of numerous genes associated with disease defense.

## Results

### Qualitative and quantitative resistance in seedling and adult plants in response to *Pgt* race TTKSK

**Figure 1:**
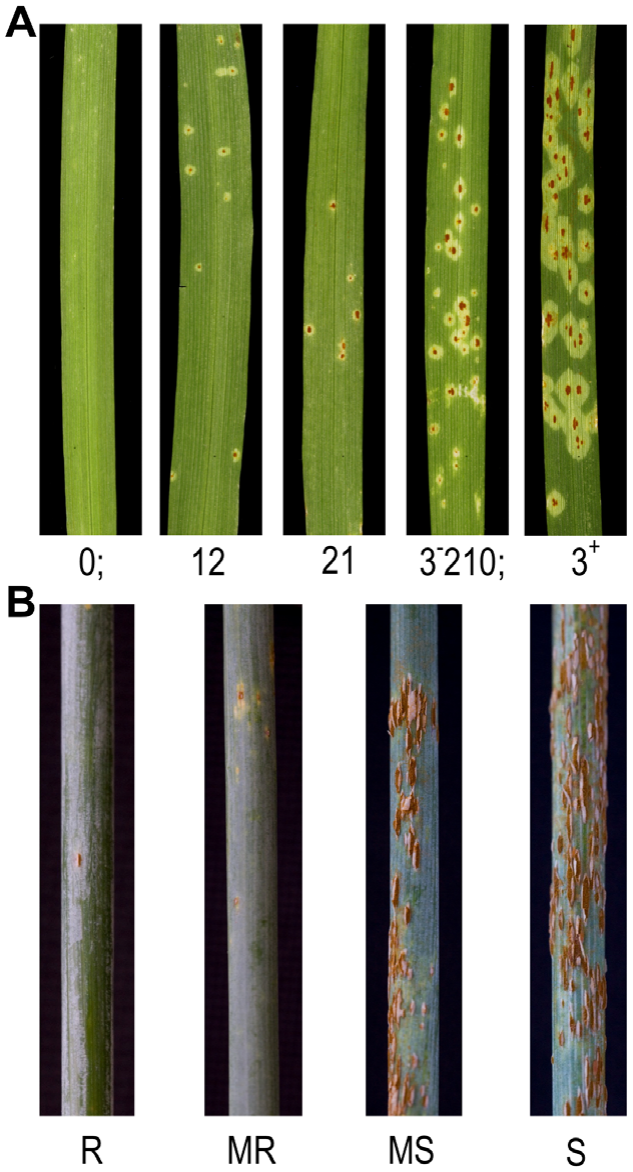
Phenotypic diversity in the barley QSM doubled haploid mapping population on first leaf in response to *Puccinia graminis* f. sp. *tritici* race TTKSK (*Pgt* race TTKSK). (A) Seedling first leaves - modified Stakman infection types reflect the size of lesions by scoring on a scale from 0 to 3+ (“;” denotes necrotic flecks) and ordered by their observed frequency. (B) Adult plants - range of lesion sizes (LES) on stems phenotyped in Njoro, Kenya during the 2008 growing season using natural inoculum of *Pgt* race TTKSK and isolates in this lineage. S  =  susceptible; MS  =  moderately susceptible; MR  =  moderately resistant; R  =  resistant.

The parents of the QSM population represent resistant and susceptible selections of barley against *Pgt* race TTKSK, with Q21861 and SM89010 exhibiting seedling infection types (IT) of 0; and 213^−^ to 3, respectively [12], [26]. As illustrated in Figure 1A, these modified Stakman IT reflect the size of lesions by scoring on a scale from 0 to 3+ (“;” denotes necrotic flecks) and are ordered by their observed frequency [12], [27], [28]. The variability of IT on SM89010 is a classic example of the mesothetic response, a phenotype frequently observed on barley when challenged with *P. graminis* [29]. This mixture of responses in SM89010 is in direct contrast to the complete resistance observed in Q21861. To identify additional loci that contribute quantitatively to resistance, we normalized the Stakman IT from the QSM population described by Steffenson and associates [12] using weighted counting of the ordered IT to generate infection frequencies, IF0, IF1, IF2, and IF3 (see Materials and Methods; Figure S1) [30]. Additionally, these infection frequencies were decomposed using principal components analysis, a method used previously to identify residual phenotypic variability in the barley Steptoe × Morex (SxM) population in response to *Pgt* race MCCF [30]. Principal component 1 (PC1) explained 74.4% of the phenotypic variance, with PC2, PC3, and PC4 explaining 15.1%, 8.7%, and 1.9% of the remaining variance, respectively.

We used a QSM genetic map generated from transcript-derived markers (TDMs) (see Materials and Methods, Dataset S1) to perform composite interval mapping (CIM) with infection frequencies and principal components [12], [27], [28], [30]. The *Rpg-TTKSK* locus on chromosome 5H at bin 49 (5H.49) was the major qualitative locus for all infection frequencies and PC1 (Table 1). In addition, several minor effect QTL were detected for IF3, PC3, and PC4 at 7H.7, 1H.35, and 2H.60, respectively. Although significant, all three QTL contributed very little to resistance as compared to the *Rpg-TTKSK* locus. Further analysis of sub-populations fixed for resistance (*Rpg-TTKSK*) or susceptibility (*rpg-TTKSK*) did not identify any other loci that substantially explained residual variation (Tables S1 and S2).

**Table 1:** Seedling resistance QTL to *Pgt-*TTKSK identified using the QSM DH lines under controlled conditions.

Trait	Chr^a^	Bin	cM		EWT^b^	LOD	AEE^c^	PVE^d^
Infection Frequency for Infection Type 0	5H	49	146.78		3.22	23.49	0.33	62.1
Infection Frequency for Infection Type 1	5H	49	146.78		3.28	18.43	0.23	53.0
Infection Frequency for Infection Type 2	5H	48	145.43		3.05	4.47	-0.12	19.6
Infection Frequency for Infection Type 3	5H	49	146.78		3.30	25.75	-0.46	60.4
	7H	7	11.18		3.30	3.98	0.10	4.3
Principal Component 1	5H	49	146.78		3.40	34.29	0.63	66.0
Principal Component 2	none detected							
Principal Component 3	1H	35	125.14	3.01		3.20	-0.09	13.6
Principal Component 4	2H	60	193.54	3.27		5.50	-0.10	24.8

a Chromosome.

b Experiment-wise threshold determined through CIM that included re-selection of background markers for each of 1,000 permuted versions of the phenotype data.

c Additive effect estimate expressed in trait units; positive values indicate the Q21861 allele causes an increase.

d Phenotypic variance explained by the allelic difference at the test locus, expressed as a percentage of total variance.

**Figure 2:**
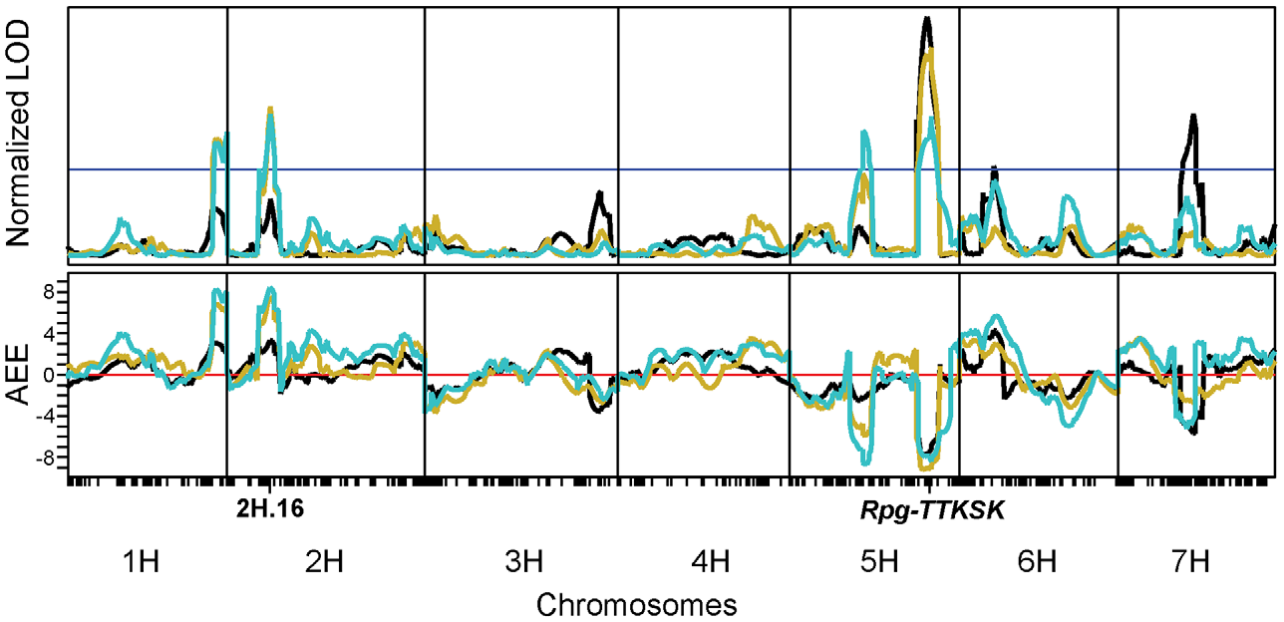
Field evaluation of the QSM population in Njoro, Kenya, identifies two quantitative trait loci on chromosomes 2H and 5H that contribute significantly to resistance. Upper panel, the LOD curves from composite interval mapping of the *Pgt* race TTKSK infection coefficients [product of percent severity and lesion size (scale of 0.25–1.0)] in the 2008 growing season. Black, gold, and cyan correspond to the 7 Oct 2008, 17 Oct 2008, and 7 Nov 2008 phenotype collection dates, respectively. LOD curves were normalized to their respective experiment-wise thresholds, shown as the horizontal blue line. Lower panel, the AEE for the additive effect estimate of the infection severity, with the red line denoting zero. Negative values indicate that resistance is contributed by the Q21861 allele, and positive values indicate that resistance is contributed by the SM89010 allele.

In parallel with the seedling experiments, infection phenotyping was performed in Njoro, Kenya during the 2008 season using natural inoculum of *Pgt* race TTKSK and isolates in this lineage. Reactions of QSM progeny were assessed three times in October and November by estimating the severity of rust infection (SEV; scale from 0 to 100%) on stem and leaf sheath tissue and also lesion size on a semi-quantitative scale (LES; scale from 0.25 to 1.00) (Figure 1B). An additional trait termed the ‘infection coefficient’ (IC), was generated by multiplying percent rust infection by the numeric code for uredinia size (SEV × LES). As shown in Table 2 and Figure 2, resistance was predominantly mediated by *Rpg-TTKSK* (contributed by the Q21861 allele), with negative additive effect estimates (AEE) of SEV by 7.7%, LES by 0.1 units, and IC by 8.4% (*i.e.*, a reduction in disease). The second most prevalent QTL identified among the set of temporal observations was located on chromosome 2H at bin 16 (2H.16). This 2H.16 locus (contributed by the SM89010 allele) had negative AEE of SEV by 6.7%, LES by 0.1 units, and IC by 8.5%. QTL analysis using resistant and susceptible QSM sub-populations (based on their *Rpg-TTKSK* allele) revealed that resistance mediated by 2H.16 was only detectable in the resistant sub-population. A two-way ANOVA test also revealed a significant interaction term between 5H.49 and 2H.16, further implicating 2H.16 as an enhancer of *Rpg-TTKSK*.

**Table 2:** Adult plant resistance QTL identified using the QSM DH lines grown in a 2008 field trial in Njoro, Kenya.

Trait	Date	Chr^a^	Bin	cM	EWT^b^	LOD	AEE^c^	PVE^d^
Severity	7-Oct-08	5H	46	141.4	3.26	9.03	-7.17	27.3
		7H	32	76.8	3.26	6.04	-5.72	15.7
	17-Oct-08	1H	47	165.6	3.22	4.38	6.18	10.2
		2H	16	44.2	3.22	4.02	5.88	9.3
		5H	27	74.9	3.22	5.77	-7.72	14.1
		5H	46	141.4	3.22	7.14	-7.95	17.6
	10-Nov-08	2H	16	44.2	3.30	3.92	7.09	11.0
		5H	45	141.3	3.30	5.26	-8.34	15.5
		6H	14	33.8	3.30	3.68	6.80	10.3
Lesion Size	7-Oct-08	3H	53	178.2	3.14	4.03	-0.08	11.2
		5H	46	141.4	3.14	11.04	-0.14	35.2
	17-Oct-08	2H	16	44.2	3.15	4.84	0.07	11.3
		3H	53	182.2	3.15	4.57	-0.07	10.9
		5H	25	72.2	3.15	6.90	-0.09	10.9
		5H	50	148.1	3.15	10.02	-0.11	28.0
	10-Nov-08	5H	27	74.9	3.13	6.21	-0.06	19.3
		5H	48	145.4	3.13	4.06	-0.04	11.8
		6H	4	6.7	3.13	3.38	0.04	9.6
Infection Coefficient	7-Oct-08	5H	46	141.4	2.98	10.25	-8.14	30.9
		7H	32	76.8	2.98	6.36	-6.10	16.8
	17-Oct-08	1H	44	154.8	3.17	4.16	6.76	10.9
		2H	16	44.2	3.17	5.21	7.35	13.3
		5H	48	145.4	3.17	7.35	-9.03	19.9
	10-Nov-08	1H	47	165.6	3.21	4.33	8.04	11.7
		2H	16	44.2	3.21	3.63	7.28	9.6
		5H	27	74.9	3.21	5.75	-10.09	16.3
		5H	48	145.4	3.21	4.32	-7.83	11.5

a Chromosome.

b Experiment-wise threshold determined by composite interval mapping that included reselection of background markers for each of 1,000 permutations of the phenotype data.

c Additive effect estimate expressed in trait units; positive values indicate the Q21861 allele causes an increase.

d Phenotypic variance explained by the allelic difference at the test locus, expressed as a percentage of total variance.

QTL analysis of both seedling and adult progeny of the QSM population revealed that the most significant locus contributing to resistance was *Rpg*-*TTKSK*. In seedlings, greater than 50% of the phenotypic variance for IF0, IF1, IF3, and PC1 was attributed to *Rpg*-*TTKSK*, whereas, *Rpg*-*TTKSK* explained 11.5% to 35.2% of the phenotypic variance in adult plants surveyed under field conditions, depending on the trait and date of data collection. In the experiments involving adult plants, the weaker relative contribution of *Rpg-TTKSK* suggests that it may act so strongly in seedlings that the effect of loci such as 2H.16 may be masked (Table 1). We hypothesized that the signaling components associated with *Rpg-TTKSK*-mediated defense response could be identified by characterizing the regulation of host gene expression in seedlings of the QSM population inoculated and mock-inoculated with *Pgt* race TTKSK. To maximize the variability among experimental genotypes and treatments, we considered *Pgt* infection kinetics as well as barley-*Pgt* time-course expression profiling data [31]. We selected 24 hours after inoculation (HAI), just after formation of *Pgt* haustoria and during intracellular hyphal growth in seedlings inoculated and mock-inoculated with *Pgt* race TTKSK [10], [11]. Furthermore, this time point should capture a mixture of PTI and ETI responses, providing opportunities to assess their potential overlap in barley-stem rust interactions.

### Gene expression between the parents of the QSM population and in response to *Pgt* race TTKSK inoculation

**Figure 3:**
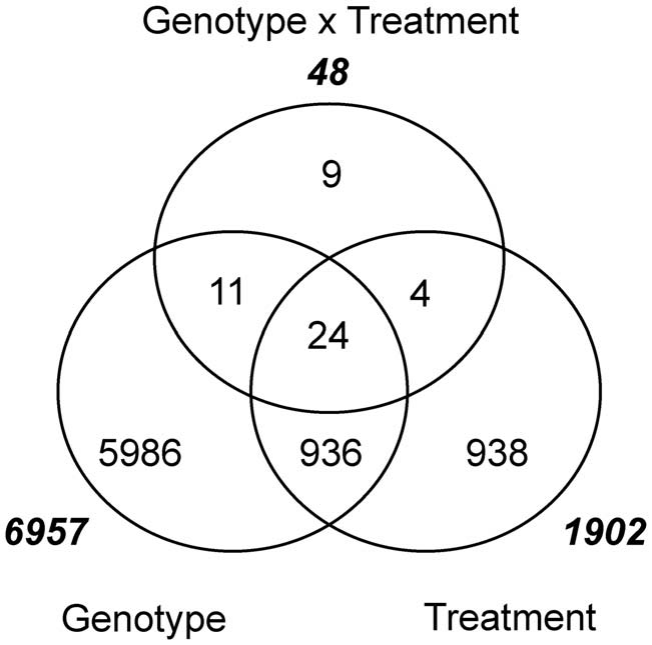
Genes differentially expressed using parents Q21861 and SM89010 for genotype, treatment (INOC versus MOCK), and their interaction. A two-way ANOVA using genotype (Q21861 and SM89010) and treatment (INOC and MOCK) was performed using the natural log normalized expression data to determine the number of differentially expressed genes between genotypes, treatments, and their interaction. False discovery rate was controlled at 5%. Totals for each class of interaction are displayed in bold italics.

Variation in gene expression between the parental lines Q21861 and SM89010 was estimated by using four biological replicates that were randomized among the 75 doubled-haploid progeny of the QSM population (see Materials and Methods). Plants were inoculated with *Pgt* race TTKSK urediniospores suspended in a light-weight mineral oil or mock-inoculated with spore-free mineral oil. For each genotype, pools of 5 first seedling leaves were harvested at 24 HAI, mRNA extracted, and hybridized to individual Barley1 GeneChips, which contain probe sets representing 22,792 genes [32]. A two-way ANOVA using genotype (Q21861 and SM89010) and treatment (INOC and MOCK) was performed using the natural log normalized expression data to determine the number of differentially expressed genes between genotypes, treatments, and their interaction. Histogram-based estimation for false discovery rate (FDR) [33] revealed 6,957, 1,902, and 48 significant differences for genotype, treatment, and genotype × treatment effects when controlled for an FDR of 5% (Figure 3). Thus, effects of polymorphisms between Q21861 and SM89010 that are independent of treatment account for the differential expression of ∼25% of the genes on the Barley1 GeneChip, or ∼78% of the total number of differentially expressed genes (Figure S2). The majority of genes with significant differences with respect to genotype had fold change less than 2 (71.6%). For the treatment effect, 1,902 genes were differentially expressed, of these, 995 were induced and 907 were suppressed. For those genes that were induced, 362 (36.4%) displayed a fold change greater than 2, while only 107 (11.8%) suppressed genes met the same 2-fold change threshold. Concordantly, the relatively small number of genes (*i.e*., 48) with an interaction between genotype and treatment was expected, as most variation in gene expression attributed to inoculation is insensitive to the genotype assayed [34], [35]. These results are similar to other plant-fungal interactions examined, where modulation of gene expression is a robust response [36], [37]. In short, if a gene is induced, it is almost always induced, or conversely, if a gene is suppressed, it is almost always suppressed in response to pathogen challenge.

### *Pgt* race TTKSK-responsive gene expression in the QSM progeny

Observation of differentially expressed genes in the parents alone sheds light on only a small fraction of the diverse genetic responses associated with defense. By contrast, the use of a segregating population provides a biallelic sampling that incorporates genotypic variability when detecting differences between treatments (INOC and MOCK). In addition, the greater number of individuals allows for a precise estimation of differential expression regardless of the allele used in our experiment [38]. Two approaches were used to estimate the difference in expression levels between INOC and MOCK in the QSM segregating population; first, by performing an ANOVA between all QSM lines in INOC versus MOCK, and second, using a paired *t*-test with respect to QSM line between INOC and MOCK.

**Figure 4:**
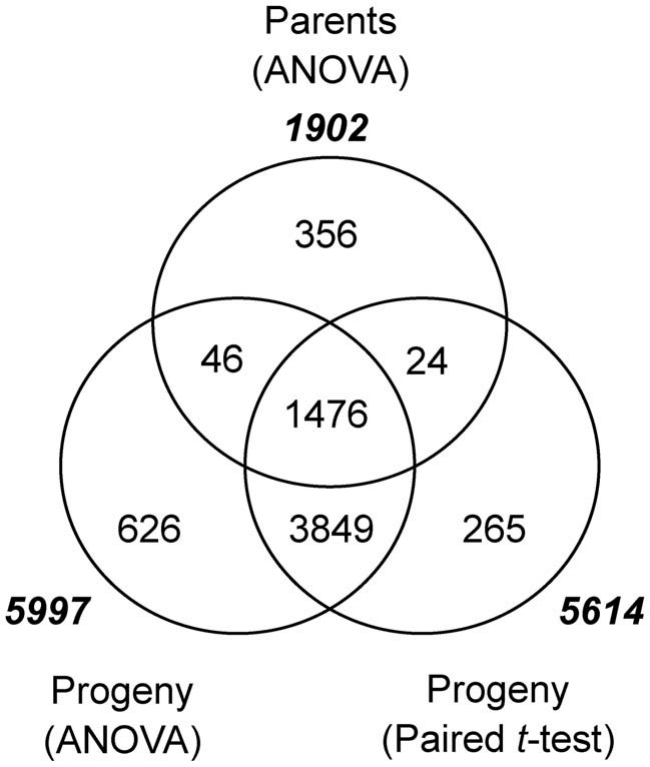
Genes differentially expressed in parents and progeny as a result of *Pgt* race TTKSK-inoculation. Differential expression in the parents was determined with a two-way ANOVA using genotype (Q21861 and SM89010) and treatment (INOC and MOCK), and controlling the false discovery rate (FDR) at 5%. Both ANOVA and paired *t*-test were used to identify differentially expressed genes in the progeny between INOC and MOCK, controlling the FDR at 0.1%. Totals for each class of interaction are displayed in bold italics.

As summarized in Figure 4, controlling the FDR at 0.1%, 5,997 and 5,614 genes were differentially expressed among progeny lines using the ANOVA and paired *t*-test approaches, respectively, with an overlap of 5,325. This suggested that the difference found between INOC and MOCK either by pooling lines (ANOVA) or between paired lines (paired *t*-test) was consistently detected as responsive to inoculation. A significant overlap was found between genes that were differentially expressed in the progeny (5,325) and those that were differentially expressed in the parents (1,902), with the intersection consisting of 1,476 genes (Figure 4). Though highly overlapping, these two gene lists had a considerable number of genes not shared in the intersection (3,849 for the progeny; 426 for the parents). We found that this was mainly accounted for by the higher sensitivity to declare differential expression when using progeny as compared to parents, as the correlation of log-fold change of genes differentially expressed in the parents or DH progeny but not both was *r* ^2^ = 0.83. These results indicate that a considerable proportion of the barley transcriptome is reprogrammed by 24 HAI in response to *Pgt* inoculation.

### Identification of eQTL in *Pgt* race TTKSK INOC and MOCK treatments

The substantial genotypic variability between Q21861 and SM89010, paired with the strong gene expression response to inoculation with *Pgt* race TTKSK, suggests that this population is ideal for identifying the regulatory components that reprogram the defense transcriptome of barley. We identified eQTL using composite interval mapping in both INOC and MOCK experiments using natural log normalized expression data from the QSM population [39], [40]. Individual experiment-wise thresholds (EWT) were determined for each expression trait in both experiments by permuting expression values 1,000 times; the CIM analyses were performed with reselection of background markers on each permuted data set, which is the more stringent implementation of this approach [41], [42]. When controlled at α = 0.05, EWT for INOC and MOCK exhibited mean LOD EWT of 3.138 and 3.142, respectively. These are slightly lower than global LOD EWT estimated from 1,000 random probe sets (INOC: 3.168 and MOCK: 3.167) [43], where a single threshold is used for all genes within a tissue/treatment. As shown in Table 3, at least one eQTL was detected for 13,919 and 15,468 expression traits in INOC and MOCK, respectively, with an intersection of 10,127 traits (Datasets S2 and S3). Estimates of FDR among expression traits that exceeded their EWTs at one or more genomic locations were 8.2% (0.05×22,792/13,919) and 7.4% (0.05×22,792/15,468) for INOC and MOCK, respectively.

**Table 3:** Number of significant eQTL per trait detected by composite interval mapping for the INOC and MOCK experiments.

Number of eQTL detected per expression trait	Number of expression traits	
	INOC	MOCK
1	8,988	9,181
2	3,815	4,674
3	975	1,385
4	130	209
5	10	18
6	1	1

**Figure 5:**
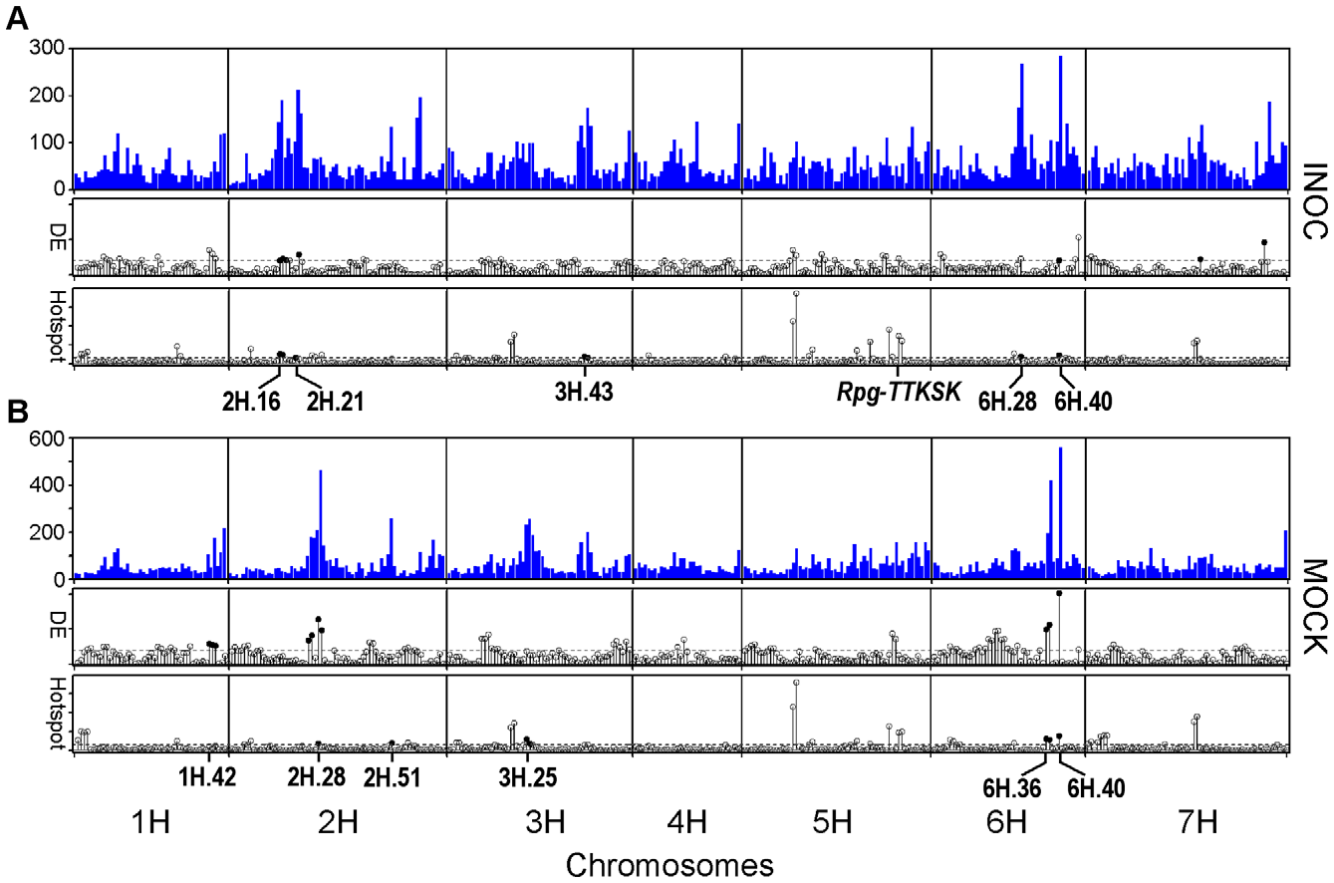
Distribution of eQTL, hotspots, and differentially expressed genes across the QSM genetic map in INOC (*Pgt* race TTKSK) and MOCK experiments. (A) Top: Histogram of eQTL in the INOC experiment. Middle: Log_10_ of the contingency χ^2^ *p*-value for regions over- or under-sampled for genes with eQTL differentially expressed in response to *Pgt* race TTKSK, as illustrated by closed and open circles, respectively, above the threshold cutoff of *p* = 0.001 (dashed line). Bottom: Log_10_ of the contingency χ^2^ *p*-value showing regions containing a significant proportion of genes either over or under-represented. Positive (hotspots) and negative (coldspots) are illustrated by closed and open circles, respectively, above the threshold cutoff of *p* = 0.001 (dashed line). (B) Ordered similarly to (A) showing data for the MOCK experiment.

The frequency of eQTL that met the EWT for both treatments is shown across the genetic map in Figure 5. eQTL were found to be unevenly distributed across the genetic map, with several regions appearing to contain either an excess or shortage of eQTL (hotspots and coldspots, respectively). Hotspots may coincide with a greater density of genes (e.g., a genomic region with little recombination, common in pericentromeric regions [44]) or even by the occurrence of a regulator of steady-state mRNA levels with strong allelic variation. Sequencing of the 5-Gb barley genome is still underway [45], thus, we could not directly compare all eQTL to their physical position or the specific number of genes within each bin. As an alternative, TDMs have been applied as surrogates for the physical positions of genes as a means to estimate the number of genes located within a chromosomal region [46]. Regions over and under-saturated with eQTL can be determined by using a contingency χ^2^ test on the ratio of TDM:eQTL as compared to the entire experiment. As shown in Figure 5 and Table 4, we identified five non-overlapping regions in MOCK and five non-overlapping regions in INOC oversaturated with eQTL with *p*<0.001. Except for the shared hotspot 6H.40, all of these are distinct, indicating that *Pgt* race TTKSK elicits extensive remodeling of transcriptional regulation.

**Table 4:** Bins on the genetic map that are significantly over-saturated with eQTL or inoculation responsive genes in INOC and MOCK experiments.

Data Set	Saturation Term	Chromosome	Bins	-log_10_(*p*-value)
INOC	DE^1^	2H	16	2.04
			17	2.24
			18	2.07
			22	2.86
		6H	40	2.01
		7H	36	2.23
			56	4.56
	eQTL	2H	16	4.87
			17	4.62
			21	3.10
		3H	43	3.70
			44	3.00
		6H	28	3.80
			40	4.48
MOCK	DE	1H	42	2.91
			43	2.72
			44	2.61
		2H	25	3.34
			26	4.09
			28	6.34
			29	4.77
		6H	36	4.93
			37	5.59
			40	10.09
	eQTL	2H	28	3.72
			51	3.83
		3H	25	5.81
			26	3.34
		6H	36	6.08
			37	5.72
			40	7.68

1 Number of differentially expressed genes in INOC versus MOCK comparison (n = 369).

### Patterns and inheritance of gene expression in the *Rpg*-*TTKSK* region

We hypothesized that an eQTL hotspot would form at the *Rpg-TTKSK* locus. However, this region was significantly under-saturated for eQTL in INOC (-log_10_(*p*)  = 14.62). Alternately, the *Rpg-TTKSK* locus could impart resistance by modulating the expression of a small set of genes at 24 HAI. We identified 88 genes with *Pgt* race TTKSK-specific regulation at the *Rpg-TTKSK* locus (5H.48/49/50), with several genes known to function in PTI, ABA signaling, and reorganization of the actin cytoskeleton (Table S3). In parallel to *de novo* regulation, regulatory perturbation at *Rpg-TTKSK* may manifest itself in the strengthening or weakening of basal expression after inoculation with *Pgt* race TTKSK. We found seven such cases among the 46 genes with eQTL at the *Rpg-TTKSK* locus (Table S4); for Contig4389_at, Contig4391_at, Contig7092_s_at, Contig7641_at, Contig9278_at, Contig26405_at, and rbah27g12_at, the AEE for the eQTL differed by more than 0.10 when the strengths of the effects were compared between INOC and MOCK. Of particular interest was the probe set Contig7092_s_at, as it hybridizes to *Adf3* [17], a gene that was previously implicated in INOC-specific regulation at the *Rpg-TTKSK* locus with the probe set Contig7093_at. Contig7093_at has AEE of 0.90 contributed by the SM allele in INOC, whereas Contig7092_s_at has AEE of 1.51 contributed by the SM allele in MOCK as compared to an AEE of 1.80 in INOC. This strong allele-dependent expression of *Adf3* in INOC was confirmed by probe level analysis. We sequenced *Adf3* in Q21861, SM89010, and 12 DH progeny (6 Q allele and 6 SM allele). This analysis revealed that probes 1–5 of Contig7092_s_at and probes 1–3 of Contig7093_at contained no SNPs between the probe source sequence (cv. Morex) and all other lines examined above. Analysis of these monomorphic probes verified the true expression level polymorphism as opposed to sequence-dependent hybridization efficiency. Note: Since the *Rpg5* sequence was not identified prior to chip design, we were therefore unable to assay its expression for this analysis.

### Identification of regions oversaturated for *Pgt* race TTKSK-responsive genes

Phenotypic QTL analysis of seedling and adult progeny implicated several resistance factors distinct from *Rpg*-*TTKSK*. These loci may represent additional basal defense regulators, such as PRR-mediated recognition of PAMPs that alter the expression of genes involved in non-specific resistance [47]. To assess evidence for this type of regulation, we identified regions that were oversaturated for genes that are differentially expressed between *Pgt* race TTKSK and mock-inoculation. We used the 5,997 genes identified as differentially expressed between INOC and MOCK treatments of the QSM progeny (Figure 4) and applied a contingency χ^2^ test on the ratio of genes with eQTL that are differentially expressed as compared to the total number of genes with eQTL at each bin (5,997:22,792 gene; 26.31%). As shown in Figure 5, five non-overlapping regions in both MOCK and INOC were significantly oversaturated for genes with eQTL that are also differentially expressed, inoculation-responsive genes (*p*<0.01; Table 4). Of particular interest were regions 2H.28/29 in MOCK and 2H.16/17/18, 2H.21/22, and 6H.40 in INOC, as they also were oversaturated with eQTL within their respective experiments.

### Dissecting the regulatory hierarchy of the 2H.16 *trans*-eQTL hotspot

**Figure 6:**
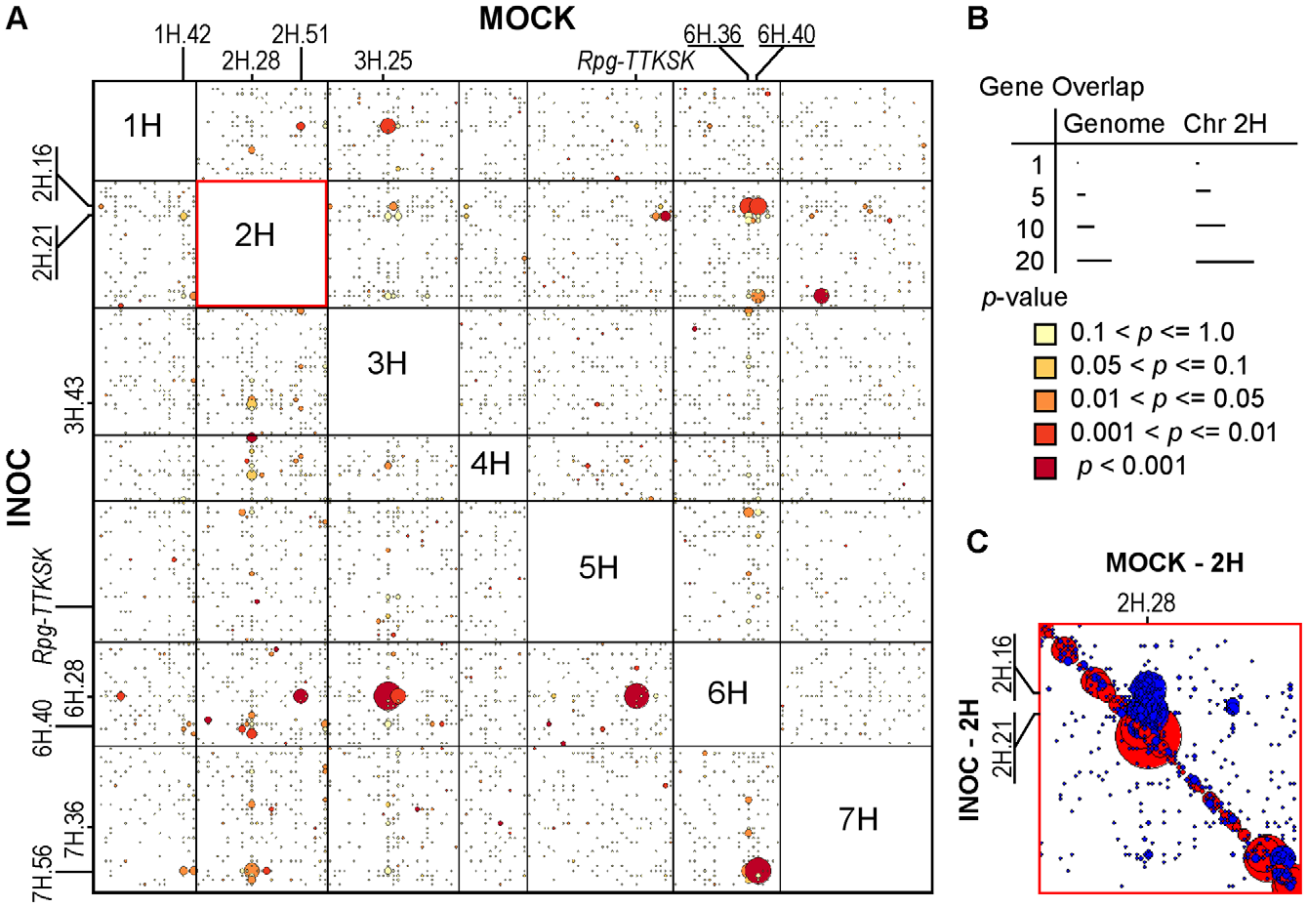
Genes from 2H.28/29, 3H.27, 6H.36/38, and 6H.40 loci are regulated by the 2H.16 *trans*-eQTL hotspot after challenge by *Pgt* race TTKSK. (A) Genes with eQTL on different chromosomes in INOC and MOCK experiments are included in the larger panel (5,538 genes), where the genetic positions of the most significant eQTL (based on LOD score) for each experiment is shown (see legend in panel B). The horizontal and vertical axes correspond to INOC and MOCK, respectively, and where each position in the plot is a superbin. (B) Gene overlap is visualized based on the diameters of circles, showing the number of genes regulated for a given region between the two experiments (smallest to largest representing 1 to 16 genes). Bootstrap-determined *p*-values are represented with colors based on the significance of the number of genes overlapping between INOC and MOCK under a random distribution, excluding eQTL that reside on the same chromosome. (C) Individual bin position of the most significant eQTL in INOC and MOCK on chromosome 2H, where a significant distortion from the *cis*-diagonal implicates altered regulation between the MOCK 2H.28/29 locus and INOC 2H.16/17/18 and 2H.21/22 loci. Filled red circles with diameter corresponding to the number of genes with the peak of their maximum eQTL shared between INOC and MOCK. If a position is off-diagonal, it is a filled blue circle with similar correspondence to the number of genes.

Hundreds of genes come under new regulation at the 2H.16 *trans*-eQTL hotspot as a result of inoculation with *Pgt* race TTKSK. By comparing the positions of the most significant eQTL for each gene in the INOC and MOCK experiments, we asked whether genes regulated at this locus are specific to pathogen-induction, or alternatively, if they are regulated by different loci between MOCK and INOC. We then determined if an overlap between two loci was significant between INOC and MOCK by generating a bootstrap *p*-value based on the number of genes shared under a random distribution and excluding comparisons between *cis*-chromosomal positions. As shown in Figure 6, 5,538 of 10,127 genes (54.7%) have their most significant eQTL on different chromosomes between INOC and MOCK experiments. The altered regulation of eQTL between INOC and MOCK was not evenly spread across chromosomes, but instead was saturated at several vertical and horizontal positions in the map, relative to MOCK and INOC, respectively. Several of the saturated regions with *p*<0.001 (dark red circles in Figure 6A) coincided with eQTL hotspots, either with loci that were significantly over-saturated with eQTL in MOCK but not INOC (2H.28, 2H.51, 3H.25/26, and 6H.40), the reverse (2H.21, 6H.28, 6H.40), or loci not associated with a saturation in eQTL. Many of these genes have alternative regulation at the sites of eQTL hotspots, but others are distributed throughout the genome. The only eQTL hotspot to be shared between treatments was 6H.40. However, the composition of 6H.40 is significantly altered, suggesting a reprioritization in the genes regulated by this locus. The eQTL hotspots at 3H.43/44 and 6H.28 in INOC form after inoculation without any apparent saturation from MOCK loci. In addition, some loci (e.g., 2H.59/60, 7H.56/57) were found to regulate a significant number of genes as a result of inoculation, but were not found to be oversaturated with eQTL in INOC. All the phenomena described above demonstrate the complexity introduced by challenge with *Pgt* race TTKSK, where transcriptional reprogramming is modulated by the activation, deactivation, or reprioritization of regulatory loci that affect transcription.

### The 2H.16 *trans*-eQTL hotspot functions as a master switch that responds to pathogen attack

The barley transcriptome undergoes significant reprogramming in response to environmental stimuli, such as cold [48], salinity [49], drought [50], or pathogen stress [36], [51]. A marked example of this reprogramming is the transfer of regulatory control from several distinct loci in MOCK to the 2H.16 locus in INOC, wherein a total of 368 genes come under alternate regulation after challenge by *Pgt-TTKSK* (Table S5). As shown in Table 5, significant loci that met the bootstrap *p*-value cutoff of 0.05 were 6H.40, 6H.36/37, 1H.1/2/3/4, 3H.27, and 7H.37/38, transferring regulation of 10, 10, 3, 5, and 3 genes to the 2H.16 locus after inoculation with *Pgt* race TTKSK, respectively. Exclusion of eQTL on the same chromosome may have removed loci that are genetically distinct; therefore we used both manual identification and *cis*-chromosome bootstrap *p*-values to identify 2H.28/29 as one additional MOCK locus where regulatory control was transferred to 2H.16 in INOC.

**Table 5:** Loci significantly over-saturated for a transfer in regulation to the 2H.16 *trans*-eQTL hotspot after inoculation with *Pgt* race TTKSK.

Locus		Number of genes			
MOCK	INOC	Maximum LOD eQTL^a^	All eQTL^b^	Bootstrap *p*-value	Correlation of AEE (r^2^)
2H.28/29	2H.16	22	42	<0.001	0.97
6H.40	2H.16	10	25	0.003	-0.98
6H.36/37	2H.16	10	23	0.005	-0.97
3H.27	2H.16	5	10	0.040	-0.86
7H.37	2H.16	3	3	0.040	NA
1H.1/2/3/4	2H.16	3	3	0.016	NA

a Maximum LOD eQTL refers to the expression QTL with the most significant association with the locus described in each experiment (MOCK or INOC).

b All eQTL are considered that are regulated in MOCK and INOC experiments.

Furthermore, we found that the overall extent of eQTL migration was underestimated, as our analysis of alternative regulation between INOC and MOCK focused on maximal eQTL (Table 5). As such, 42, 25, 23, 10, 3, and 3 genes are regulated at the MOCK loci 2H.28/29, 6H.40, 6H.36/37, 3H.27, 1H.1/2/3/4, and 7H.37, respectively, with the inclusion of non-maximal eQTL. Of the six loci that significantly contribute to the INOC 2H.16 locus, 1H.1/2/3/4, and 7H.37 lacked an increase in eQTL when including non-maximal eQTL and were excluded from further analysis. In addition to the genes regulated in MOCK by these six loci, the INOC 2H.16 locus regulates the expression of an additional 199 genes with MOCK eQTL distributed across the genetic map and 73 genes that did not have a detectable eQTL in MOCK.

### Coordinated reprogramming of inoculation-dependent regulons

**Figure 7:**
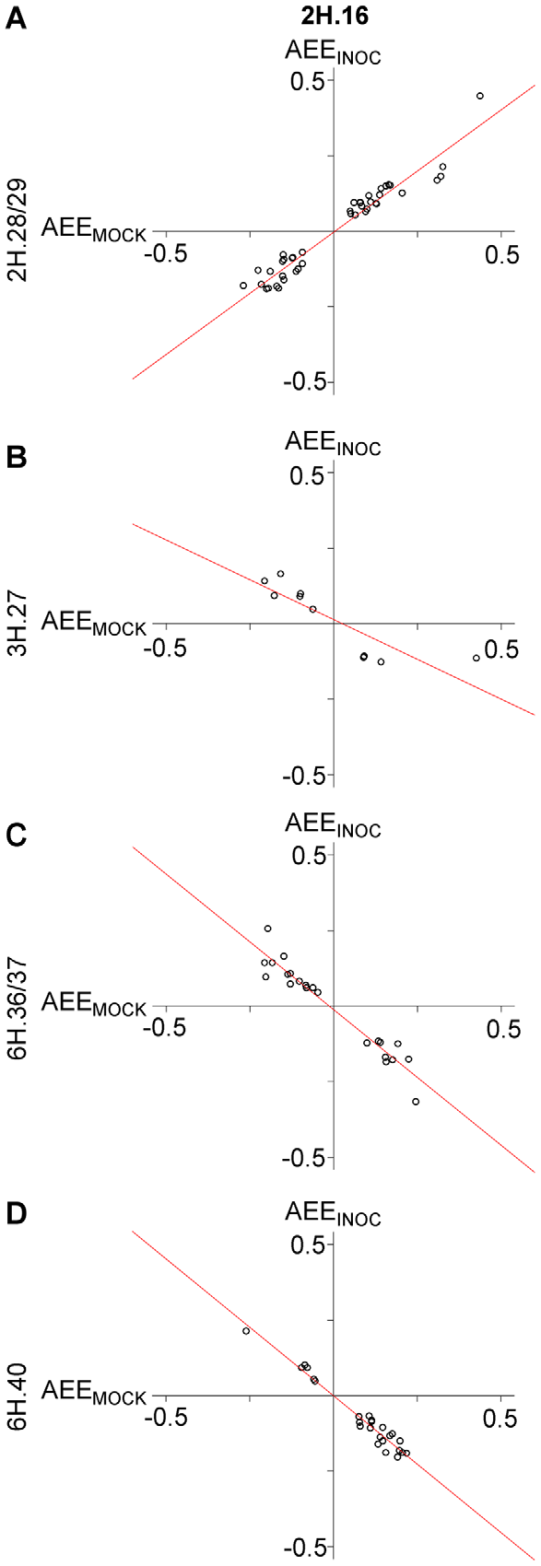
Allelic effects in gene expression are predictive between INOC and MOCK treatments by distinct loci. Plots comparing the additive effect estimate (AEE) of the INOC eQTL at 2H.16 locus with the AEE of the MOCK eQTL at (A) 2H.28/29, (B) 3H.27, (C) 6H.36/37, and (D) 6H.40. A positive value for AEE implies that if a plant carries the Q21861 allele, then gene expression is increased by twice the estimate for that allelic effect. Conversely, negative values for AEE means the SM89010 allele increases the level of expression.

Alternate transcriptional control in the INOC and MOCK experiments suggested that one or more regulator(s) at 2H.16 in INOC override the regulation exerted by MOCK loci 2H.28/29, 3H.27, 6H.36/37, 6H.40, as well as at additional loci distributed across the genome. This coalescence of regulation in INOC suggests that each of the loci identified in MOCK may itself coordinate the expression of a distinct regulon. If this were true, one might expect the functional polymorphisms that underlie these eQTL to act pleiotropically on their respective targets in similar ways. To test this, we compared AEE for eQTL between INOC 2H.16 and the four MOCK loci listed in Table 5. As illustrated in Figure 7, the parent that increased gene expression was either conserved (2H.28/29) or reversed (3H.27, 6H.36/37, 6H.40) for each MOCK locus as compared to the INOC 2H.16, with correlations of *r* ^2^ = 0.97, −0.86, −0.97, and −0.98, respectively. This predictive power between MOCK and INOC regulatory loci suggests that these genes represent four inoculation-dependent regulons. These regulons were not entirely distinct, as seven genes were shared between 2H.28/29 and 6H.40, and one gene was shared between 2H.28/51 and 6H.36/37, 2H.28/29 and 3H.37, and 3H.27 and 6H.36/37. The overlap in control among regulons is correlated with the number of genes regulated by each locus and is likely an underestimate due to population size. Thus, coordinated reprogramming from multiple loci in MOCK to the single INOC locus suggests that these genes belong to a buffered regulatory complex in mock-inoculated leaves that is consolidated by a master switch in response to pathogen infection.

**Figure 8:**
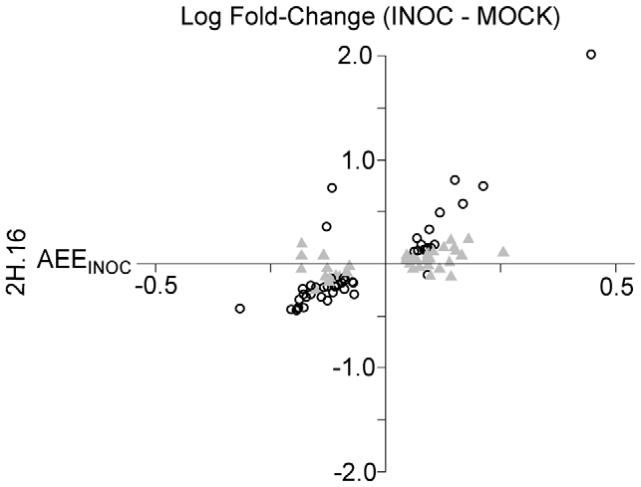
Allelic effects for INOC-specific eQTL are significantly correlated with the fold change between *Pgt* race TTKSK-inoculated and mock-inoculated plants. Plot comparing the natural logarithm of the fold change difference between INOC and MOCK versus the AEE from the INOC eQTL at 2H.16. Genes included have predictive AEE between 2H.16 in INOC and 2H.28/29, 3H.27, 6H.36/37, and 6H.40 in MOCK (Figure 5). Black circles correspond to genes declared differentially expressed between INOC and MOCK populations, grey triangles correspond to those genes that did not meet the 0.1% FDR cutoff.

Coupled with the dependence on alternative regulation after challenge with *Pgt* race TTKSK, over-saturation of inoculation-responsive genes at the 2H.16 locus implies that this locus largely determines the extent to which a gene is differentially expressed between INOC and MOCK. When we considered the AEE in INOC eQTL at the 2H.16 locus, we found that it was highly predictive of the direction of differential expression between INOC and MOCK, with 78 of 90 genes affirming this association (Figure 8). Additionally, significant correlation was observed between the AEE in INOC and the log-fold change of differential expression (*r* ^2^ = 0.71; 90 genes) (Figure 8). Selecting only those genes differentially expressed in the comparison of INOC versus MOCK strengthened the correlation, *r* ^2^ = 0.85 (48 genes). In contrast, genes not declared differentially expressed between treatments had a considerably weaker correlation of *r* ^2^ = 0.51 (42 genes). These results indicate that the regulation in INOC from the 2H.16 locus is either the principal source or major component of gene expression changes due to challenge by *Pgt* race TTKSK.

### The SM89010 allele at the 2H.16 locus leads to transcriptional suppression of defense-associated genes

Our final analyses aimed to characterize shared aspects of the genes regulated by the 2H.16 *trans*-eQTL hotspot following challenge by *Pgt* race TTKSK. First we examined the AEEs of these genes and observed a bias in the directionality of effects. Specifically, of the 229 genes with eQTL that have positive AEE (Q21861 allele), 162 are induced and 67 are suppressed. The opposite is seen for the 139 genes with eQTL that have negative AEE (SM89010 allele), with 39 induced and 100 suppressed. Thus, for the 368 genes regulated, the SM89010 allele at 2H.16 attenuates expression levels for 262 of them (∼71%).

Since we previously established that the genes regulated by the 2H.16 *trans*-eQTL hotspot are oversaturated for differential expression in response to stem rust (Figure 5 and Figure 6), we wished to consider any additional evidence for their functional involvement in defense. To address this, we performed gene ontology (GO) enrichment analysis using the suite of analysis tools from agriGO to identify functional conservation [52]. Singular enrichment analysis (SEA) of genes having an eQTL where the SM89010 allele contributes the greater allelic effect identified over-representation of genes targeted to the plastid (37 of 111 annotated genes; *p* = 2.0e-6; *q* = 1.2e-4). In contrast, no significant GO terms were identified using SEA for eQTL with Q21861 allele contributing the greater allelic effect.

Although SEA directly tests for enrichment of GO terms, it does not take into account the magnitude of differential expression or allelic effects. To address this, we used a parametric analysis of gene set enrichment (PAGE) to incorporate these effects. In agreement with the results from SEA, we found that localization to the plastid was significant for genes down-regulated after challenge by *Pgt* race TTKSK (*p* = 1.7e-3; *q* = 3.3e-2). We also found that genes with negative allelic estimates, where the SM89010 allele results in lower expression, were moderately over-represented among the set of genes predicted to be localized to the plastid (*p* = 6.3e-3; *q* = 0.12). Additional statistically significant GO terms were identified with PAGE that were associated with either up-regulation or down-regulation after inoculation with *Pgt* race TTKSK. GO terms associated with lower expression in INOC (down-regulation) included: biosynthetic processes (GO:0009058), nitrogen compound metabolism (GO:0006807), nucleobase, nucleoside, nucleotide and nucleic acid metabolism (GO:0006139), and cellular biosynthetic processes (GO:0044249). In contrast, protein metabolism (GO:0019538), protein modification (GO:0006464), macromolecule modification (GO:0043412), cellular protein metabolism (GO:0044267), and localization to the membrane (GO:0016020) are associated with up-regulated genes (greater expression in INOC).

The relative enrichments of GO terms observed are diagnostic of plant-pathogen interactions where genes involved in protein metabolism, modification, and membrane localization are typically up-regulated, while those genes targeted to the plastid are down-regulated [53]–[55]. Together with the directionalities of allelic effects, we have shown that the prototypical pattern of gene expression associated with defense is attenuated by the SM89010 allele at the 2H.16 *trans*-eQTL hotspot. This result is paradoxical, since presence of SM89010 alleles across this same locus also enhances *Rpg-TTKSK*-mediated resistance (Table 2).

## Discussion

The intricacies of genetic inheritance of gene expression have contributed significantly to our current understanding of gene regulation [18], [21], [56], [57]. Early studies established that the majority of expression polymorphisms were highly heritable and provided evidence for both monogenic and oligogenic regulation of gene expression [21]. Additionally, the majority of eQTL appeared to act locally, such that eQTL are localized near the physical position of the gene. These initial observations looked to be conserved across eukaryotes, although this may reflect the ease of detecting *cis*-eQTL as compared with *trans*-eQTL [21]–[23]. In line with this observation, local regulation generally has a stronger effect as compared to distant regulation. Underlying each *trans*-eQTL is one or several functional polymorphism(s) that typically manipulates the expression of tens, if not hundreds, of genes. Thus, the weaker effect of *trans*-eQTL is compensated for by the co-localization of many genes and is identified by the occurrence of *trans*-eQTL hotspots. Notably, eQTL analysis of segregating populations of Arabidopsis and barley has been used to establish links between circadian rhythm and metabolism [58], determine the limited pleiotropic effect of mutation on gene expression [59], and identify candidate genes for disease resistance QTLs based on *cis*-eQTL [51], [60].

### What forms the basis for qualitative resistance?

The presence of *trans*-eQTL hotspots at the site of major regulators has become a common theme in the control of gene expression [18], [61], [62]. Interestingly, a hotspot was not identified at the *Rpg-TTKSK* locus, but several models can account for its absence. First, our selection of 24 HAI may represent a very early (or late) time point in the activation of resistance signaling, such that only the primary targets of *Rpg-TTKSK*-specified resistance would be differentially regulated at this time. Second, if the primary transcriptional targets of *Rpg-TTKSK*-mediated resistance had considerable structural variation in their promoters, then the regulatory contribution from the *Rpg-TTKSK* locus may be effectively masked by strong *cis*-eQTL effects, or be too small to be detected with this approach. In this scenario, *trans*-eQTL hotspots composed of secondary targets will form at the genetic positions of primary transcriptional targets of *R*-gene signaling, rather than the *R* gene itself. These two hypotheses overlap, as the selection of time points would determine whether primary, secondary, or more general responses are detected. Lastly, the primary resistance response mediated by *Rpg-TTKSK* may not include gene expression as a causal component in defense.

The absence of a *trans*-eQTL hotspot refocused our efforts to identify *cis*-eQTL that are biologically relevant at the *Rpg-TTKSK* locus, an approach used previously to dissect partial resistance to barley leaf rust [51]. ADF proteins are involved in the reorganization of the actin cytoskeleton by altering the rate of actin dissociation from the pointed ends of actin filaments [63] and are known to play a role in basal defense and *mlo*-mediated resistance in barley-powdery mildew interactions [64]. Notably, *Adf3* is located within 5H.49 and is proximal to the *Rpg5* resistance gene. Previously, this gene was excluded as a candidate for *Rpg5*, which mediates resistance to *Pgs* isolate 92-MN-90, because the ADF3 amino acid sequence was identical between resistant and susceptible cultivars [17]. Our results suggest that after inoculation with *Pgt* race TTKSK, *Adf3* has enhanced regulation (AEE_INOC_ > AEE_MOCK_) at 5H.49, with lower expression in lines carrying *Rpg-TTKSK*. Therefore, resistance is associated with the suppression of *Adf3* expression, a hypothesis suggested for *Adf2* by Brueggeman and colleagues [17]. Although *Adf3* was not considered a candidate for *Rpg5* based on the failure to observe non-synonymous variation between Q21861 and susceptible alleles of the coding region [16], our data suggest that *Adf3* may be a factor contributing to *Rpg-TTKSK*-mediated resistance based on its strong expression polymorphism. Several hypotheses have been put forth for the biological role of ADF in plant-pathogen interactions [16], [30]. Here, the enhanced expression of *Adf3* in plants carrying the susceptible (SM89010) allele implicates its functional role as a susceptibility factor induced by *Pgt* race TTKSK. Significant structural variation does occur in the promoter of *Adf3* [16], although additional functional analysis will be required to establish the requirement of *Adf3* induction in compatibility. However, it has been shown that an intact host actin cytoskeleton is required for successful colonization in several plant-fungal pathosystems [65], therefore *Adf3* may be a potential target of pathogen-derived effectors.

### What accounts for massive transcriptional reprogramming in response to stem rust?

Even in the absence of a *trans*-eQTL hotspot at *Rpg-TTKSK*, extensive transcriptome reprogramming due to invasion by *Pgt* race TTKSK revealed key regulators that were altered between INOC and MOCK treatments. Most significant was the *trans*-eQTL hotspot at 2H.16, where saturation in both the number of eQTL and inoculation-responsive genes suggests that regulator(s) at this locus usurp the steady-state regulatory machinery to actively remodel gene expression. Dissection of this hotspot revealed a transcriptional hierarchy of several distinct loci in MOCK that converge on 2H.16 after challenge with *Pgt* race TTKSK, with a predictive allelic effect between INOC and MOCK conditions.

The co-localization of the hotspot with an enhancer of adult plant, *R* gene-mediated defense may implicate a causal relationship between gene expression and enhanced resistance. There exists some difficulty in making this connection, as the cloning of both classical and expression QTL can be confounded by the presence of tightly linked genes contributing to the phenotype. In mouse, dissection of the *Qrr1* (*QTL rich region on chromosome 1*) region found that multiple genes likely regulate different subsets of *trans*-eQTL that had previously been grouped together [66]. With the use of informative recombinants and multiple populations, Mozhui and colleagues [66] separated the *Qrr1* region into proximal and distal portions, and found the distal region specifically regulated RNA metabolism and protein synthesis. In doing so, they were able to focus the eQTL candidate gene list that underlies a classical QTL associated with seizure susceptibility. This case study provides a model for how a single locus associated with an abundance of both QTLs and *trans*-eQTL was broken into two regions that are associated with entirely different pathways. Similarly, it is possible that the 2H.16 region may be comprised of several regulators. At present, our strongest evidence against this hypothesis is the predictive power in the allelic effects between the MOCK loci (2H.28/29, 3H.27, 6H.36/37, and 6H.40) and the INOC 2H.16 locus, suggesting a single regulator or family of regulators that control these regulons after challenge with *Pgt* race TTKSK.

### The deactivation of the MOCK 2H.28 *trans*-eQTL hotspot in INOC

**Figure 9:**
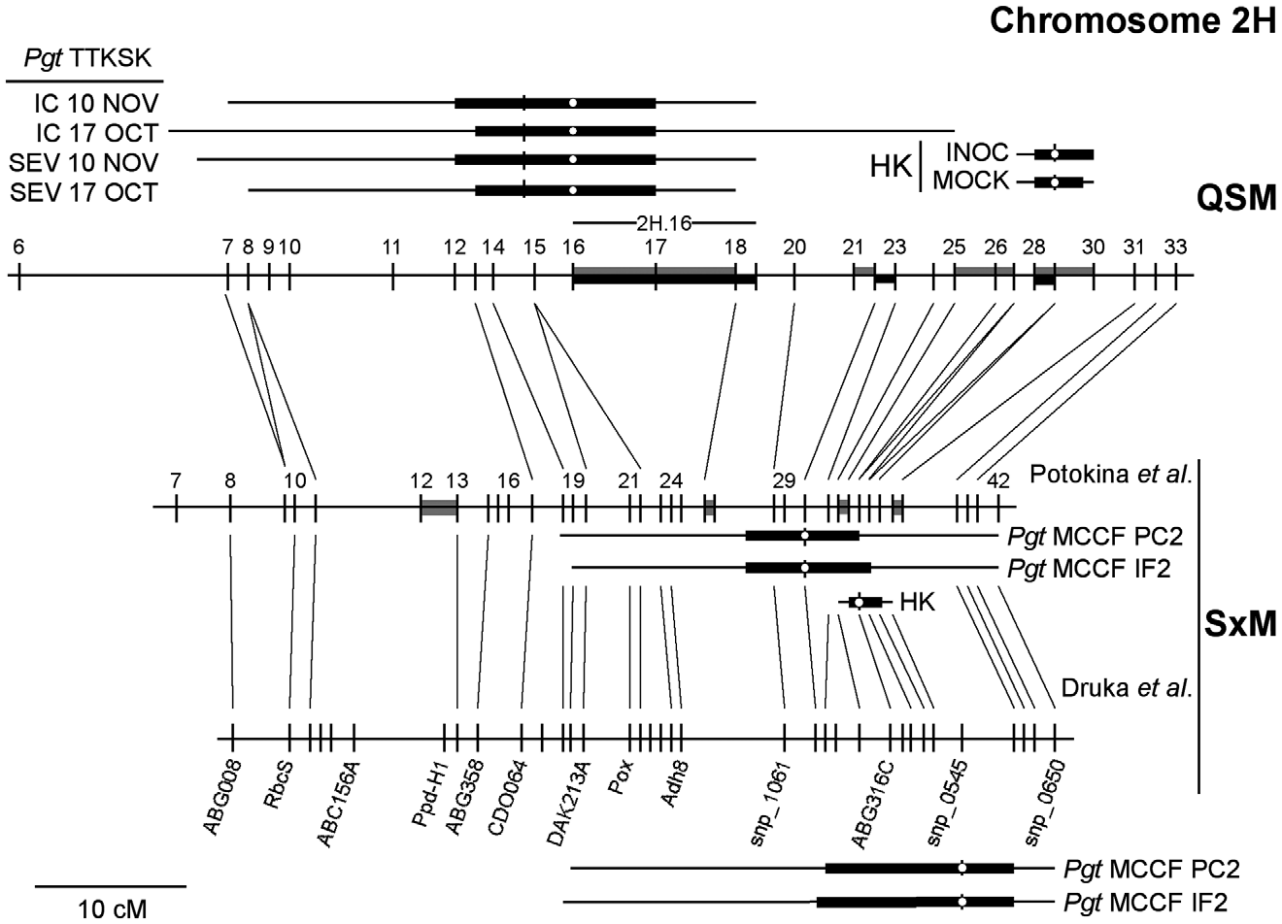
The deactivation of the MOCK 2H.28 *trans*-eQTL hotspot in INOC. The QSM and SxM TDM genetic maps (upper and middle panels) were linked by shared TDMs, shown as lines between these panels. Numbers indicate the bin number for each genetic map. One and 2-LOD support intervals derived from standard interval mapping are shown for all QTL reported with box and whiskers, respectively. HK is the sensory transduction histidine protein kinase (represented by probe set Contig13680_s_at). White circles are the peak position identified using composite interval mapping. Phenotypic QTLs for the adult QSM field trials in Njoro, Kenya are shown above the QSM genetic map. Two genetic maps exist for the SxM population, one derived from TDMs (Potokina map; middle panel) and another from RFLP, SNP, AFLP, and RAPD markers (Druka map; lower panel). As the support intervals for QTL were considerably different, we report them here showing the correspondence of these two SxM maps [30], [46]. Grey and/or black filled in regions on the genetic map indicate whether the region was oversaturated with eQTL (*trans*-eQTL hotspot) or differentially expressed genes, respectively.

In our investigation of eQTL regulation after treatment with *Pgt* race TTKSK, we focused on the dynamics in response to pathogen invasion. This is one approach for identifying genes that act as nodes in regulatory networks, but several other methodologies may be similarly powerful. For example, candidate genes can be identified from unrelated eQTL experiments by using additional information such as physical map position, functional annotation, expression polymorphisms, and correlation [18], [51], [67]. Druka and colleagues [30] provided a case study for the eQTL candidate gene selection of the cloned resistance gene *Rpg1* [68] by using correlation and tissue-specific expression to associate the causal gene, albeit from unrelated tissue (grain) [30]. Similarly, they extended this approach to identify candidate genes for several minor effect stem rust resistance QTLs from the SxM population. They leveraged expression profiling of *rpr1* [69], a gene required for *Rpg1*-mediated resistance, and physical map position to identify a sensory transduction histidine protein kinase (represented by probe set Contig13680_s_at) that was strongly down-regulated in non-inoculated *rpr1* plants and physically mapped near the *QPgt.StMx-2H* QTLs (IF2 and PC2) [30]. In light of the results from this previous study and the importance of both phenotypic QTL and *trans*-eQTL hotspots on chromosome 2H in our work, we linked the QSM and SxM genetic maps via the conserved TDMs used to generate both maps (Figure 9). Based on shared TDMs, it appears that the *QPgt.StMx-2H* QTLs detected in the SxM population inoculated with *Pgt* race MCCF and the QTL identified in adult QSM progeny in Njoro, Kenya are largely distinct. Both QTLs have broad 2-LOD support intervals that overlap, but the 1-LOD support intervals are separate. Though the histidine protein kinase does have an overlapping 1-LOD support interval with *QPgt.StMx-2H*, we found that the peak of the *QPgt.StMx-2H* co-localized precisely with the 2H.21 and 2H.22 regions, shown by our results to be a *trans*-eQTL hotspot and over-saturated with differentially expressed genes after inoculation with *Pgt* race TTKSK. The histidine protein kinase exhibits a strong expression level polymorphism in the QSM population similar to the SxM population. An eQTL for the gene co-localizes with the 2H.28/29 *trans*-eQTL hotspot and is detected in both INOC and MOCK, having a LOD of 16.71 (15.24) and AEE of 0.67 (0.66), with greater expression contributed by SM89010 allele in INOC (MOCK). Thus, three tightly linked loci, 2H.16, 2H.21/22, and 2H.28/29 control the QTLs to *Pgt* race TTKSK, *Pgt* race MCCF, and the regulation of a steady-state *trans*-eQTL hotspot, respectively. It is of interest that both QTLs detected in the SxM and QSM populations against *Pgt* races TTKSK and MCCF, respectively, enhance the effect of their respective *R* genes, *Rpg-TTKSK* and *Rpg1*. As Druka and colleagues used tissue derived from germinating embryos, it is unclear whether a *trans*-eQTL hotspot underlies the histidine protein kinase in leaf tissue in the SxM population [30]. Clearly, this region of chromosome 2H is a hotbed of phenotypic and expression QTLs that are involved in resistance to stem rust and points to an interconnected set of regulatory loci that link these genetic loci with resistance.

### Can suppression of certain defense-associated genes actually benefit overall defense?

It is interesting that the *trans*-eQTL hotspot at 2H.16 regulates genes that are both induced and suppressed in response to *Pgt* race TTKSK invasion. Overall, the allelic effects for *trans*-eQTL at this locus were biased for greater expression when carrying the Q21861 allele (304 Q21861 vs. 219 SM89010). This effect was mutually predictive with up-regulation in response to *Pgt* race TTKSK associated with the Q21861 allele. In contrast, enhancement of *Rpg-TTKSK*-mediated adult plant resistance was associated with the SM89010 allele. This is especially relevant, as Q21861 contributes *Rpg-TTKSK* and SM89010 is susceptible to *Pgt* race TTKSK. Taken together, these results suggest that transcriptional suppression was correlated with resistance, where enhancement would have been expected. This model of host-mediated gene suppression may be a defense mechanism against pathogen-mediated gene activation, which has been observed in several phytopathosystems as a method to distract or enhance accessibility of the host. The bacterial pathogen *Pseudomonas syringae* pathovar *tomato* DC3000 produces the jasmonic acid-mimic coronatine that induces jasmonic acid/ethylene-associated pathways that compete with bacterial defense pathways dependent on salicylic acid signaling [70]–[72]. In contrast, direct binding to host promoters by TAL effectors in *Xanthomonas* spp. activate genes involved in host susceptibility [73]–[76]. Though counter-intuitive, several systems have shown that this mechanism is a *bona fide* approach for manipulating the host and enhancing virulence. Therefore this locus may provide a degree of insensitivity to effector-dependent manipulation of the host.

It is important to recognize that genes regulated at 2H.16 in INOC were not dependent on *Rpg-TTKSK*. Therefore the enhanced resistance conferred by 2H.16 in the presence of *Rpg-TTKSK* suggests that the manipulation of gene expression may only impact the interaction of barley and *Pgt* under the appropriate conditions. Here, regulation at 2H.16 in INOC overrides the control of several steady-state regulators in MOCK. Thus, these MOCK regulators may be prone to manipulation by *Pgt*, whereas 2H.16 is non-responsive. Alternatively, the 2H.16 locus may control the precise timing of gene expression, such that the full impact of these genes is maximized to strengthen *Rpg-TTKSK*-mediated resistance. Ultimately, increased resolution in the 2H.16 region will be required to dissect the causal polymorphisms that enhance *R* gene-dependent adult plant resistance and the regulator(s) that generate the *trans*-eQTL hotspot.

## Materials and Methods

### Seedling resistance assays to *Pgt* race TTKSK

The barley QSM doubled-haploid mapping population was generated from a single Q21861 × SM89010 F_1_ plant [26], [77]. All data used for infection type analysis are derived from Steffenson *et al*. [12]. Briefly, three to five seeds of each doubled-haploid line or parent were planted in plastic cones and placed in flats in a completely randomized design. Plants were placed in the greenhouse at 22°C with supplemental lighting by 1,000-W sodium vapor lamps for 14 hours per day at the USDA-ARS Cereal Disease Lab, University of Minnesota, St. Paul. *Pgt* race TTKSK isolate 04KEN156/04 was initially increased on a susceptible wheat host, collected, desiccated, and stored in tubes at −80°C. Nine days after sowing (PO:0007094 - first leaf unfolded), flats were inoculated with a low density of *Pgt* race TTKSK urediniospores (0.004 mg/plant) suspended in a lightweight mineral oil carrier using the inoculation protocols described by Sun and Steffenson [29]. After inoculation, the plants were placed in a mist chamber for 16 hours in the dark, followed by light for 5 hours, and then moved to the greenhouse using the previously described conditions. Plants were phenotyped at 14 to 17 days after inoculation. The full experiment was repeated twice, with the evaluation of three to five plants per replicate.

### Adult resistance assays to *Pgt* race TTKSK

Field trials were carried out at the Kenya Agricultural Research Institute in Njoro, Kenya during the 2008 growing season. QSM DH lines were planted in 0.3 m rows (20–35 seed per row) in a completely randomized design with one replicate. Parents were included at random in the planting plan in three replicates. Infection phenotypes were scored on 7 October 2008, 17 October 2008, and 10 November 2008. The majority of lines were at the mid-dough stage of development (Zadoks scale 8.5; Feeke's scale 11.2) at the first scoring date [78], [79]. Other lines with later maturity reached the mid-dough stage by 17 October and 10 November. The bulk of the natural inoculum found in the field was typed to *Pgt* races TTKSK (used in seedling resistance assays) and TTKST (same virulence pattern as TTKSK with the addition of virulence for wheat stem rust resistance gene *Sr24*). *Pgt* race TTTSK (same virulence pattern as TTSKS with the addition of virulence for *Sr36*) may have been present at a low frequency.

### QTL analysis of resistance traits

Stakman infection types (ITs) for seedling plants were normalized using a modified approach that weights the counts of ordered ITs (Figure 1A and Figure S1, Table S6) [30]. Weights given were 1.0, 0.65, 0.25, and 0.1 for the 1^st^, 2^nd^, 3^rd^, and 4^th^ ordered ITs, respectively. IFs were determined by averaging weights for two replicates, where full weight is given to ITs of 0, 1, 2, and 3 or partial weights for ITs of ‘0;’, ‘1−’, ‘1+’, ‘2−’, ‘2+’, and ‘3−’. For partial weights, 70% is given to the IT shown (0, 1, 2, or 3) and 30% to the modified IT (‘+’ to the greater IT, ‘−’ to the lower IT). In the unique case of ‘3+’, a weight of 1.3 was given to IT 3. For adult plants, LES was quantified on a scale from 0.25 to 1.0 based on resistance or full susceptibility, where resistant is equal to 0.25, moderately resistant is equal to 0.5, and a fully susceptible LES score is 1.0 (Figure 1B). The IC was determined by multiplying the SEV by LES. Principal components analysis for seedling and adult phenotypic data was performed using R (www.r-project.org) (Table S7). Composite interval mapping (**Zmapqtl**; model 6) was performed with QTL Cartographer v1.17j, with a walking speed of 2 cM, window size of 10 cM, and five background markers (**SRmapqtl**) [39]. EWT were computed using permuted data (**Prune**) with reselection of background markers (**SRmapqtl**), where each iteration maximum LOD scores were stored and after 1,000 runs the 95^th^ quantile (α = 0.05) was selected as the EWT [41], [42]. QTLs that exceeded the EWT were extracted using **Eqtl**.

### eQTL experimental design

Two flats (each flat contained 75 doubled-haploid lines + 4 replicates of each parent  = 81 cones/flat) were grown in a completely randomized design at the USDA-ARS Cereal Disease Lab, University of Minnesota, St. Paul. For the INOC flat, a higher density of *Pgt* race TTKSK urediniospores (0.25 mg/plant) was used as compared to the seedling phenotypic assay. For the MOCK flat, spore-free mineral oil was used. After inoculation, both flats were placed in the same mist chamber for 16 hours in the dark, exposed to light for 5 hours, and then moved to the greenhouse for 2 hours. Five seedlings were harvested, pooled, and placed in liquid nitrogen for each line in the population within a 1.5 hour period at 24 HAI. RNA was extracted using a hot acid-phenol protocol and RNAeasy columns (Qiagen) were used for further purification of the isolated RNA. Labeling, hybridization, washing, and scanning were performed according to standard Affymetrix protocols using the Barley1 GeneChip which contains probe sets representing 22,792 (21,439 non-redundant) genes [32] at the ISU GeneChip Facility (www.biotech.iastate.edu/facilities/genechip/Genechip.htm).

### Development of the transcript-derived marker map

At any single genetic locus, each of the 75 doubled haploid lines carries two copies of either the Q21861 or the SM89010 allele. These two genotypes can be distinguished by differential success in hybridizing RNA to Barley1 arrays, providing robust genetic markers. Transcript-derived markers were generated as described by Potokina and colleagues [46], using an implementation in Python (www.python.org). This technique identifies single feature polymorphisms (SFPs) by using individual probes on the Affymetrix Barley1 GeneChip as quantifiable measures of probe hybridization efficiency. After background correction and quantile normalization using R/Bioconductor (www.bioconductor.org), individual probe signals were separated into two distinct groups with *k*-means clustering. Goodness-of-fit using a Z-statistic found over 2,500 quality markers for the QSM genetic map. This analysis was performed separately with the INOC and MOCK data sets, and only those markers conserved between these data sets (1,503 markers) that had three (of 75) or fewer data points missing were included. A scaffold of 294 markers shared with the SxM doubled-haploid mapping population was used to place the remaining 1,200 markers [46]. Available information for the genetic positions of genes represented on the Affymetrix Barley1 GeneChip was used to confirm marker order; this included data from a recently developed SNP-derived genetic map [44]. Manual curation of marker positions was assisted with visualization of two-point marker linkages using MadMapper (Figure S3; http://cgpdb.ucdavis.edu/XLinkage/MadMapper) [46], [80]. The final map has a total of 378 unique markers (bins) with a genetic length of 1,259 cM, with an average of approximately 3.3 recombination events between bins (Figure S4, Dataset S1).

### ANOVA of inoculated and mock-inoculated Q21861, SM89010, and QSM population

ANOVA was performed with SAS v9.1 (SAS Institute Inc., Cary, North Carolina). All comparisons between these data sets were generated using Python scripts. *q*-values were determined using histogram-based estimation proposed by Mosig and colleagues [81], using the implementation by Nettleton and associates [33].

### eQTL analysis of INOC and MOCK experiments

All microarray data for eQTL analysis were normalized with the Bioconductor implementation of the MAS5.0 algorithm (www.bioconductor.org). Composite interval mapping was performed with QTL Cartographer v1.17j, using a walking speed of 2 cM, window size of 10 cM, and five background markers [39]. eQTL that exceeded individual EWT were extracted using a Python script, such that two peaks within close proximity were declared different eQTL if the distance between peaks was greater than 2 LOD [46]. Individual EWT were computed using a combination of Python scripts, bash shell scripts, and QTL Cartographer. Briefly, composite interval mapping was performed using the same criteria in the eQTL analysis except the data were permuted (**Prune**) with reselection of background markers (**SRmapqtl**) a total of 1,000 times [41], [42]. Maximum LOD scores were stored and the 95^th^ quantile (α  =  0.05) was selected as the individual EWT.

### Detection of *trans*-eQTL hotspots

The over and under-saturation of eQTL were identified using a contingency χ^2^ test on the ratio of TDM:eQTL for a region as compared to the entire experiment. To fulfill the requirements of this contingency χ^2^ test, we merged successive bins in the genetic map until the sum of observed eQTL and TDMs was greater than 73 for INOC and 85 for MOCK for each set of bins. The same bins were used to analyze both experiments by incorporating the distribution of eQTL in MOCK and INOC in parallel.

### Bootstrap analysis of eQTL migration between INOC and MOCK

A bootstrap approach was used to estimate the significance associated with alternate regulation in the INOC and MOCK data sets for genes with eQTL in both data sets, using the maximum LOD eQTL. Genetic regions were compared with non-overlapping merged bins (superbins) that were generated with a greedy approach. This approach required a minimum number of TDMs and eQTL in INOC (73 TDM and eQTL) and MOCK (85 TDM and eQTL) to be placed within a superbin. Hence, the same bins in each experiment were collected into a single superbin. This approach is similar to that used for the identification of *trans*-eQTL hotspots and regions for over-saturation of differentially expressed genes (see Results). Two strategies were used to account for (1) alternate regulation on the same chromosome and (2) regulation on different chromosomes between INOC and MOCK. For both, genes were redistributed from MOCK using probabilities determined by the distribution of eQTL in INOC based on the eQTL histogram. This was repeated 1,000 times for the maximum LOD eQTL in the overlap between INOC and MOCK. Probabilities were generated differently for the first and second strategies by including all genes with eQTL (10,127 genes) in INOC and MOCK or only those genes with maximum LOD eQTL on a different chromosome between data sets (5,538 genes). Bootstrap *p*-values were determined by comparing the observed overlap versus the 1,000 bootstrapped samples.

### Gene ontology enrichment analysis

Gene ontology enrichment analysis was carried out using agriGO v1.0β (http://bioinfo.cau.edu.cn/agriGO) [52]. Singular enrichment analysis (SEA) was performed using the default parameters, Fisher test, the Yekutieli multi-test adjustment method, a significance level of 0.05, and a minimum number of five mapped entries using the complete set of gene ontology terms. Parametric analysis of gene set enrichment (PAGE) was used with SEA default parameters, with a difference in requiring a minimum of ten mapped entries and FDR cutoff at 0.1.

### Data access

All MIAME-compliant GeneChip profiling data are available as accession BB64 at the PLEXdb expression resource for plants and plant pathogens (www.plexdb.org), accession GSE20416 at NCBI-GEO, as well as accessions GN235, GN236, GN237, GN238 at GeneNetwork (www.genenetwork.org) [82].

## Supporting Information

Dataset S1 Excel spreadsheet of the QSM genetic map. Transcript-derived marker genetic map of the Q21861 x SM89010 doubled-haploid population. Markers have been derived from probe-level data as described in the Materials and Methods. The first sheet contains the representative genetic map containing all non-redundant markers (N  =  378). The second sheet contains data for all markers (N  =  1,494).
(XLS)

Dataset S2 Excel spreadsheet of the eQTL identified in the MOCK experiment. eQTL determined to be significant based on individual EWT in the MOCK experiment as described in the Materials and Methods.
(XLS)

Dataset S3 Excel spreadsheet of the eQTL identified in the INOC experiment. eQTL determined to be significant based on individual EWT in the INOC experiment as described in the Materials and Methods.
(XLS)

Figure S1 Modified Druka *et al.* [30] procedure for processing infection type (IT) data. Stakman ITs for seedling plants were normalized using a modified approach that weights the counts of ordered ITs [30]. Weights given were 1.0, 0.65, 0.25, and 0.1 for the 1^st^, 2^nd^, 3^rd^, and 4^th^ ordered ITs, respectively. Infection frequencies (IF) were determined by averaging weights for two replicates, where full weight is given to ITs of 0, 1, 2, and 3 or partial weights for ITs of ‘0;’, ‘1-‘, ‘1+’, ‘2-‘, ‘2+’, and ‘3-’. For partial weights, 70% is given to the IT shown (0, 1, 2, or 3) and 30% to the modified IT (‘+’ to the greater IT, ‘-’ to the lower IT). In the unique case of ‘3+’, a weight of 1.3 was given to IT 3.
(TIF)

Figure S2 Ternary plot of percent effect explained by genotype (G), treatment (T), and genotype x treatment (G x T). (A) Genes shown have over 50% of their variance (from two-way ANOVA) explained by the sum of the three effects (G, T, and G x T). Point colors of magenta and green show whether or not a given gene had a transcript-derived marker, respectively. The majority of genes having TDMs are those with a significant genotype effect, suggesting strong allelic polymorphisms between parents Q21861 (Q) and SM89010 (SM). The bottom panel shows representative examples for the extremes of genotype (B), treatment (C), and their interaction (D).
(TIF)

Figure S3 Two-point locus heat map of the QSM DH genetic map. Heat map of the non-redundant QSM DH genetic map showing linkage for all marker x marker comparisons, genetic distance between markers (right), and proportion of the contributed allele (Q: red, SM:blue) for every marker (bottom).
(TIF)

Figure S4 Recombination map of the QSM DH population.
(TIF)

Table S1 Quantitative trait loci identified in the resistant sub-population (*Rpg-TTKSK*) of seedlings of the QSM DH mapping population inoculated with *Pgt* race TTKSK.
(XLS)

Table S2 Quantitative trait loci identified in the susceptible sub-population (*rpg-TTKSK*) of seedlings of the QSM DH mapping population inoculated with *Pgt* race TTKSK.
(XLS)

Table S3 Genes identified with an inoculation-specific eQTL at the *Rpg-TTKSK* locus.
(XLS)

Table S4 Genes identified with eQTL in INOC and MOCK at the *Rpg-TTKSK* locus.
(XLS)

Table S5 Genes identified with an inoculation-specific eQTL at the 2H *trans*-eQTL hotspot.
(XLS)

Table S6 Phenotypic data for seedling and adult QSM lines inoculated with *Pgt* race TTKSK.
(XLS)

Table S7 Coefficients of the principal components for QSM seedling phenotyping.
(XLS)
