# Supplementary figures and images for: Quantitative and Qualitative Stem Rust Resistance Factors in Barley Are Associated with Transcriptional Suppression of Defense Regulons
Source: PLoS Genet. 2011 Jul 28;7(7):e1002208. doi: 10.1371/journal.pgen.1002208 (PMC3145622; doi:10.1371/journal.pgen.1002208)

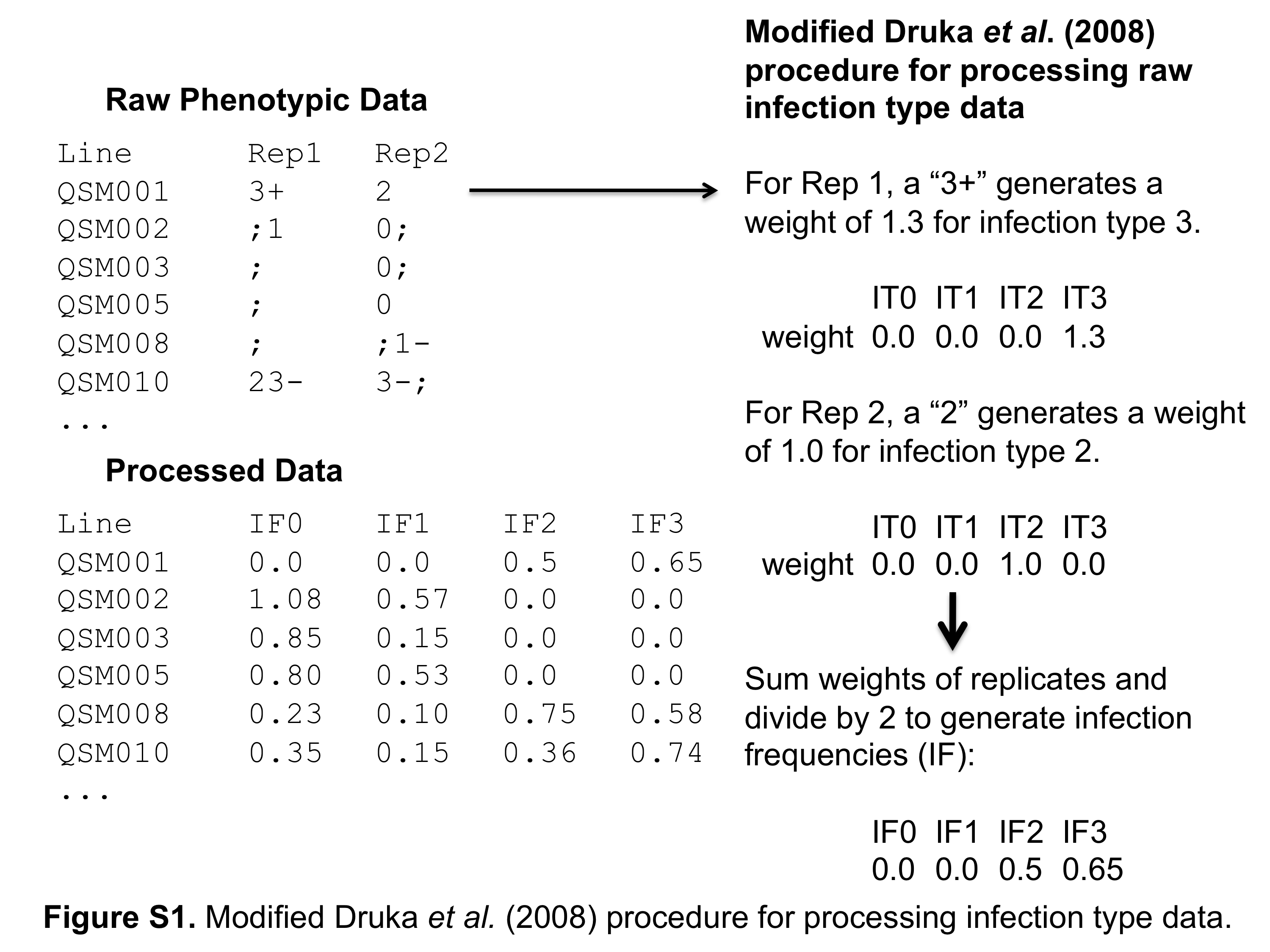

Supplement: Figure S1 — Modified Druka et al. [30] procedure for processing infection type (IT) data. Stakman ITs for seedling plants were normalized using a modified approach that weights the counts of ordered ITs [30]. Weights given were 1.0, 0.65, 0.25, and 0.1 for the 1st, 2nd, 3rd, and 4th ordered ITs, respectively. Infection frequencies (IF) were determined by averaging weights for two replicates, where full weight is given to ITs of 0, 1, 2, and 3 or partial weights for ITs of ‘0;’, ‘1-‘, ‘1+’, ‘2-‘, ‘2+’, and ‘3-’. For partial weights, 70% is given to the IT shown (0, 1, 2, or 3) and 30% to the modified IT (‘+’ to the greater IT, ‘-’ to the lower IT). In the unique case of ‘3+’, a weight of 1.3 was given to IT 3. (TIF) [file pgen.1002208.s004.tif]

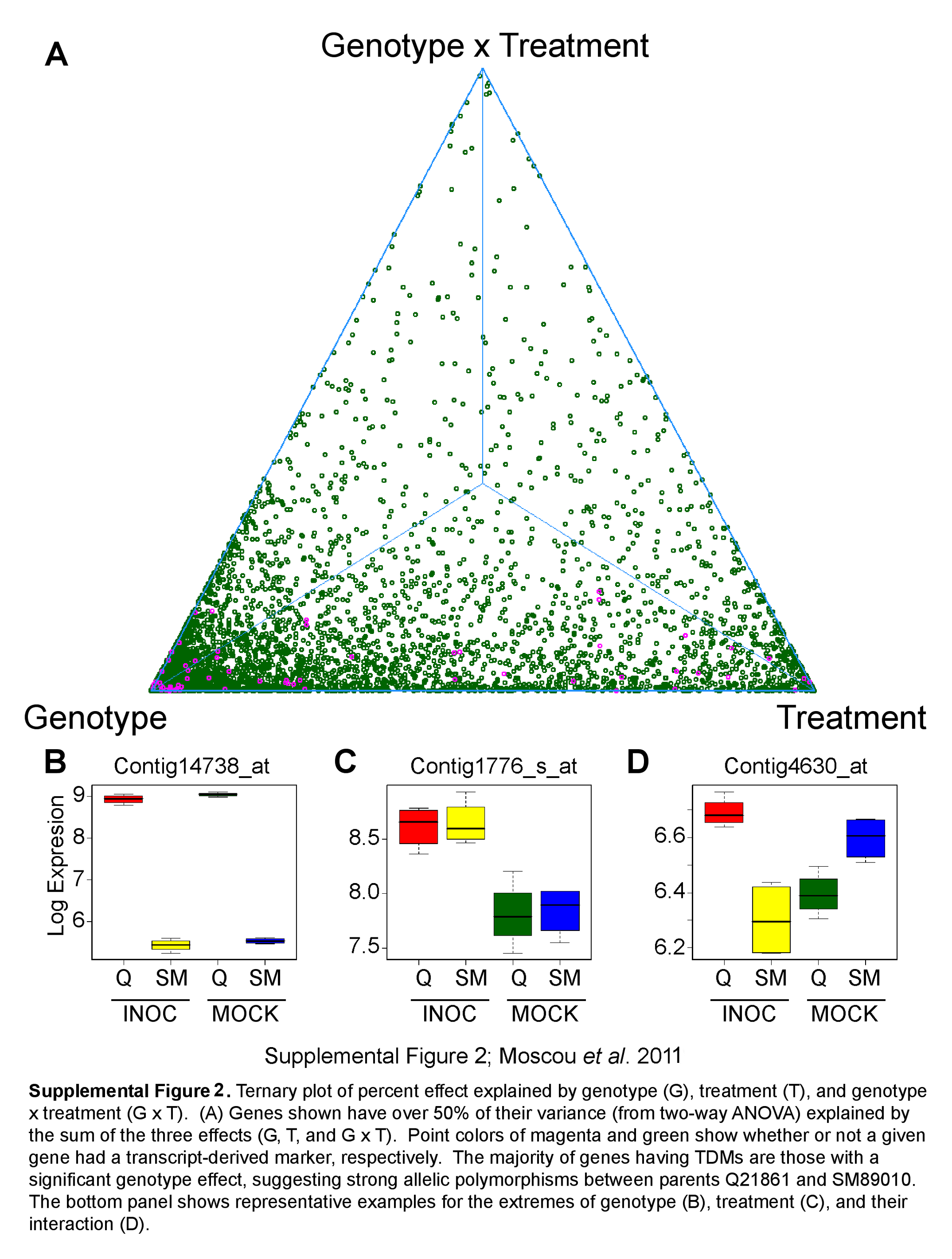

Supplement: Figure S2 — Ternary plot of percent effect explained by genotype (G), treatment (T), and genotype x treatment (G x T). (A) Genes shown have over 50% of their variance (from two-way ANOVA) explained by the sum of the three effects (G, T, and G x T). Point colors of magenta and green show whether or not a given gene had a transcript-derived marker, respectively. The majority of genes having TDMs are those with a significant genotype effect, suggesting strong allelic polymorphisms between parents Q21861 (Q) and SM89010 (SM). The bottom panel shows representative examples for the extremes of genotype (B), treatment (C), and their interaction (D). (TIF) [file pgen.1002208.s005.tif]

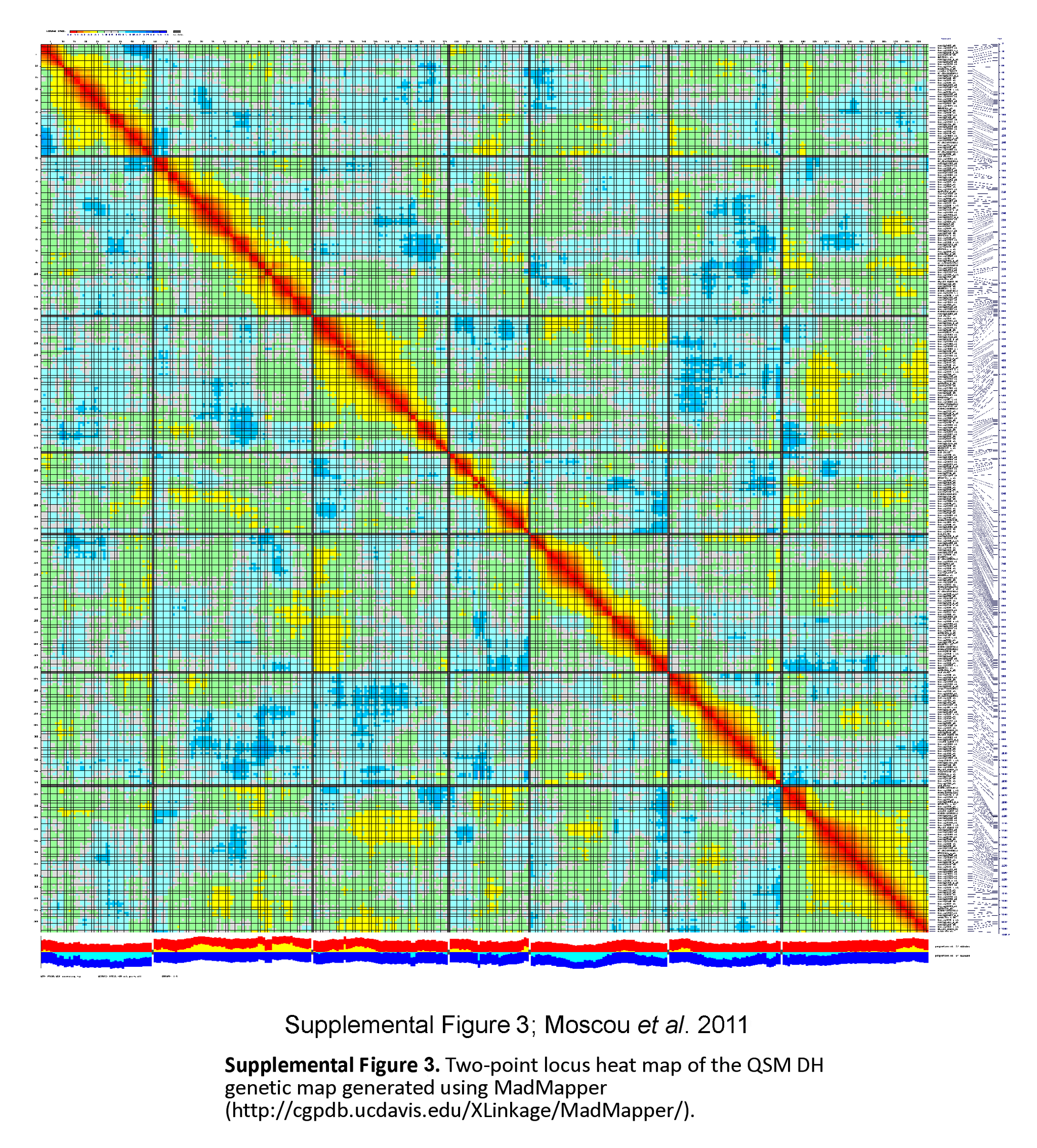

Supplement: Figure S3 — Two-point locus heat map of the QSM DH genetic map. Heat map of the non-redundant QSM DH genetic map showing linkage for all marker x marker comparisons, genetic distance between markers (right), and proportion of the contributed allele (Q: red, SM:blue) for every marker (bottom). (TIF) [file pgen.1002208.s006.tif]

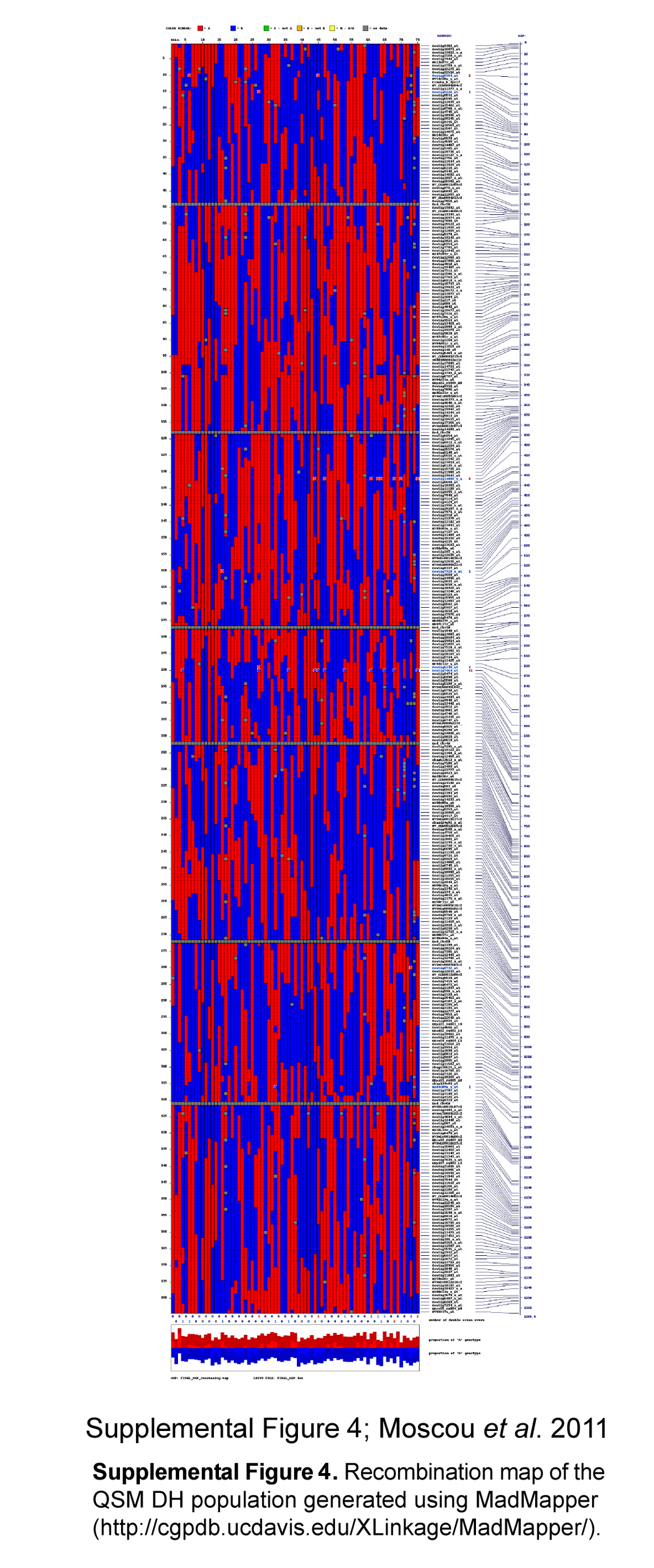

Supplement: Figure S4 — Recombination map of the QSM DH population. (TIF) [file pgen.1002208.s007.tif]
